# Supplementary material for: Simultaneous Multi-Organ Metastases from Chemo-Resistant Triple-Negative Breast Cancer Are Prevented by Interfering with WNT-Signaling
Source: Cancers (Basel). 2019 Dec 17;11(12):2039. doi: 10.3390/cancers11122039 (PMC6966654; doi:10.3390/cancers11122039)
Supplement: Supplementary file 1 [file cancers-11-02039-s001.zip › cancers-631322-suppl-final2/cancers-631322-suppl-final2.docx]

Supplemental Materials: Simultaneous Multi-Organ Metastases from Chemo-Resistant Triple-Negative Breast Cancer Are Prevented by Interfering with WNT-Signaling

Iram Fatima, Ikbale El-Ayachi, Hilaire C. Playa, Jackelyn A. Alva-Ornelas, Aysha B. Khalid, William L. Kuenzinger, Peter Wend, Jackelyn C. Pence, Lauren Brakefield, Raisa I. Krutilina, Daniel L. Johnson, Ruth M. O’Regan, Victoria Seewaldt, Tiffany N. Seagroves, Susan A. Krum and Gustavo A. Miranda-Carboni


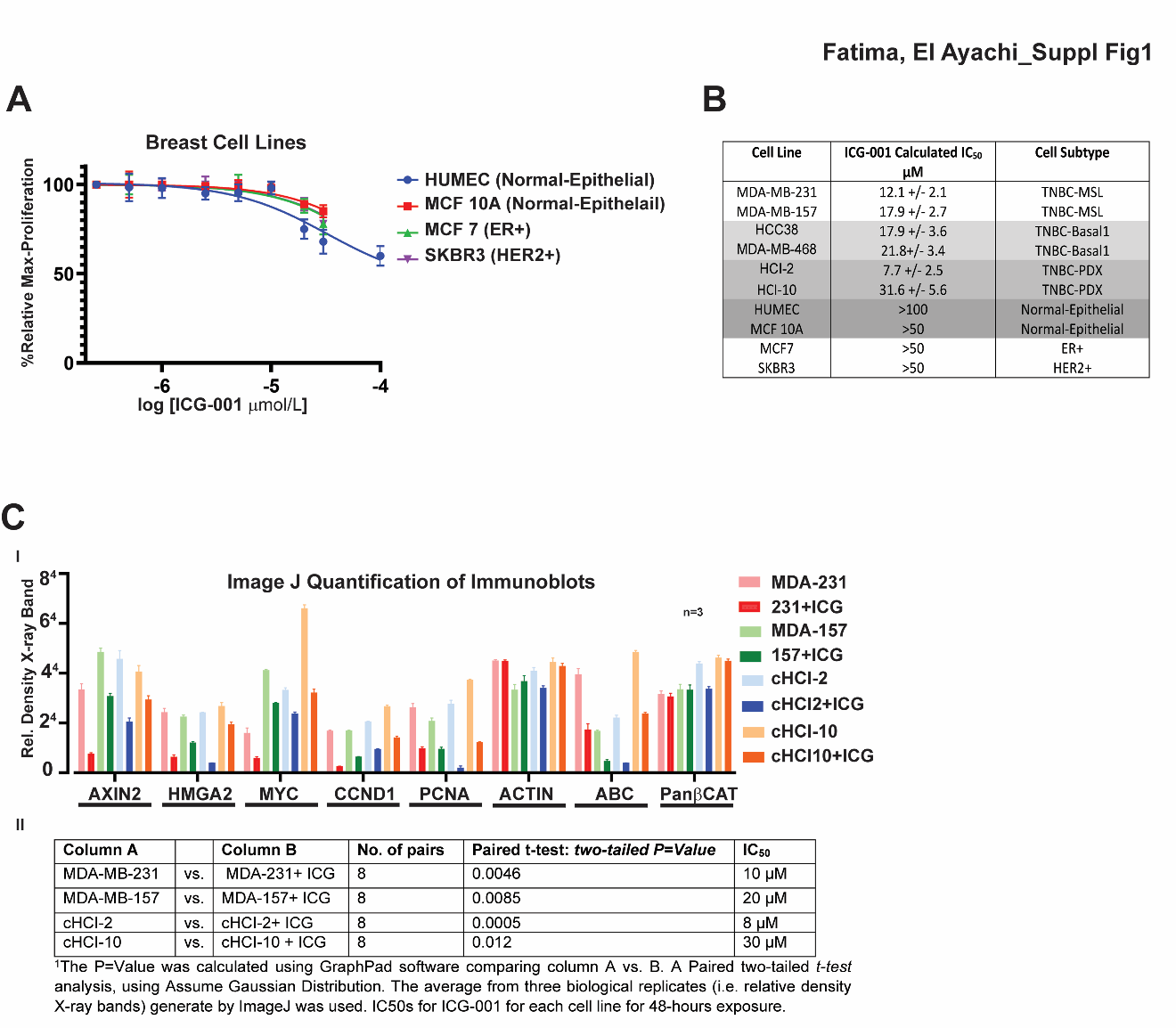


**Figure S1.** Determination of exact differential IC_50_ (DIC_50_) of ICG-001 on multiple TNBC cell lines. (**A**) WST-1 proliferation assays were conducted in human breast cancer cells lines HUMEC (normal breast epithelial), MCF10A (normal breast epithelial), MCF-7 (ER+) and SKBR3 (HER2+) that were exposed to ICG-001 at various dosages ranging from 0.2 μM–30 μM, for 48 h. (**B**) Exact IC_50_ (DIC_50_) was calculated utilizing WST-1 proliferation assays 48 h after treatment with ICG-001 at various dosages ranging from 0.2 μM-30 μM using MDA-MB-231, MDA-MB-157, HCC38, MDA-MB-468, PDX TNBC patient derived cHCI-2 and cHCI-10 cells. The control cell lines doses were from 0.2 µM-100 µM for control breast cell lines, HUMEC, MCF10A, MCF7 and SKBR3. **C** Immunoblot for AXIN2, HMGA2 MYC, CCND1, PCNA, ACTIN, non-phosphorylated Active-β-CATENIN (ABC) and total-β-CATENIN were quantified by ImageJ from biological triplicates. The average of the biological triplicates was graph as the relative density of the X-ray bands, in the absence or presence of ICG-001 at the calculated IC_50_ for each cell line for 48 h (Ci). The X-ray bands were then analyzed as paired groups of 8 within each individual cell type(s) and determine to be statistically significance, in the response to ICG-001 exposure (Cii).


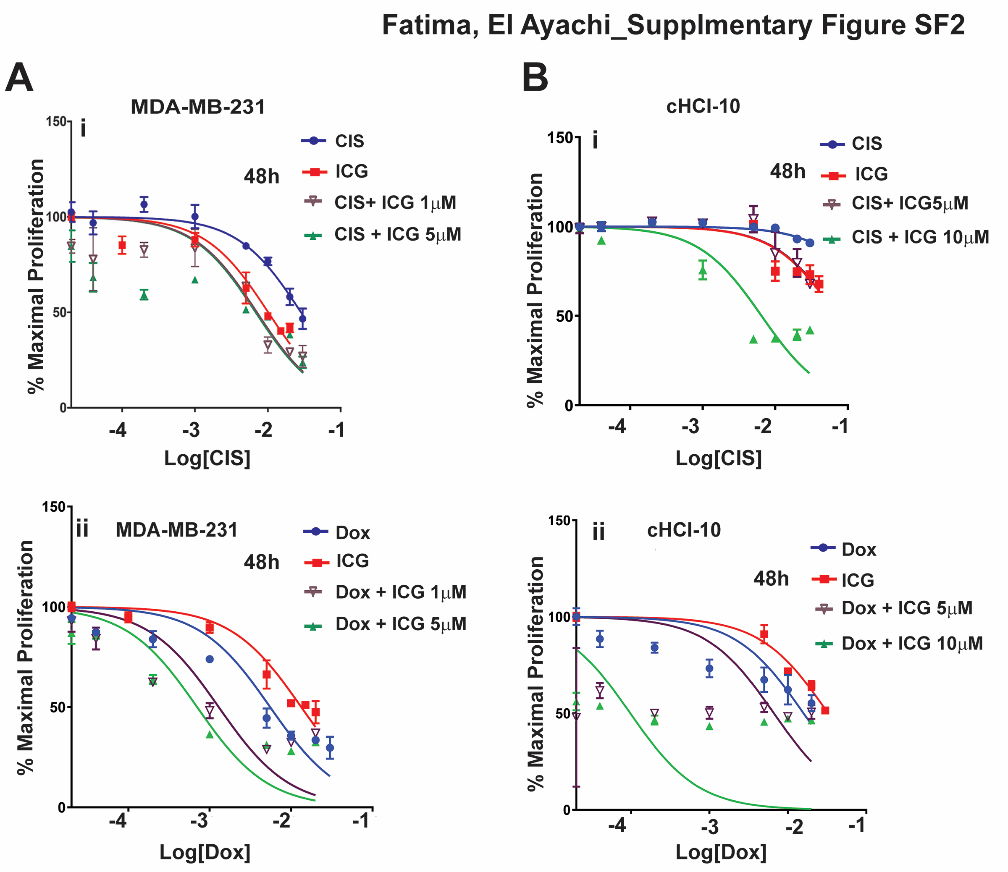


**Figure S2.** ICG-001 is able to synergize with doxorubicin *in vitro*, but not cisplatin, to repress tumor cell proliferation in the doxorubicin chemoresistant PDX-TNBC cells. MDA-MB231Luc Ai-ii and cHCI-10Luc Bi-ii cells were analyzed by WST-1 assays at 48 h, following exposure to ICG-001 (1 µM and 5 µM concentration, MDA-MB-231 or 5 µM or 10 µM concentrations for cHCI-10 cells) in combination with cisplatin or DOX at various increasing concentrations, demonstrating inhibition of tumor cell proliferation. Results are expressed as mean ± SE, from three biological replicates in triplicates.


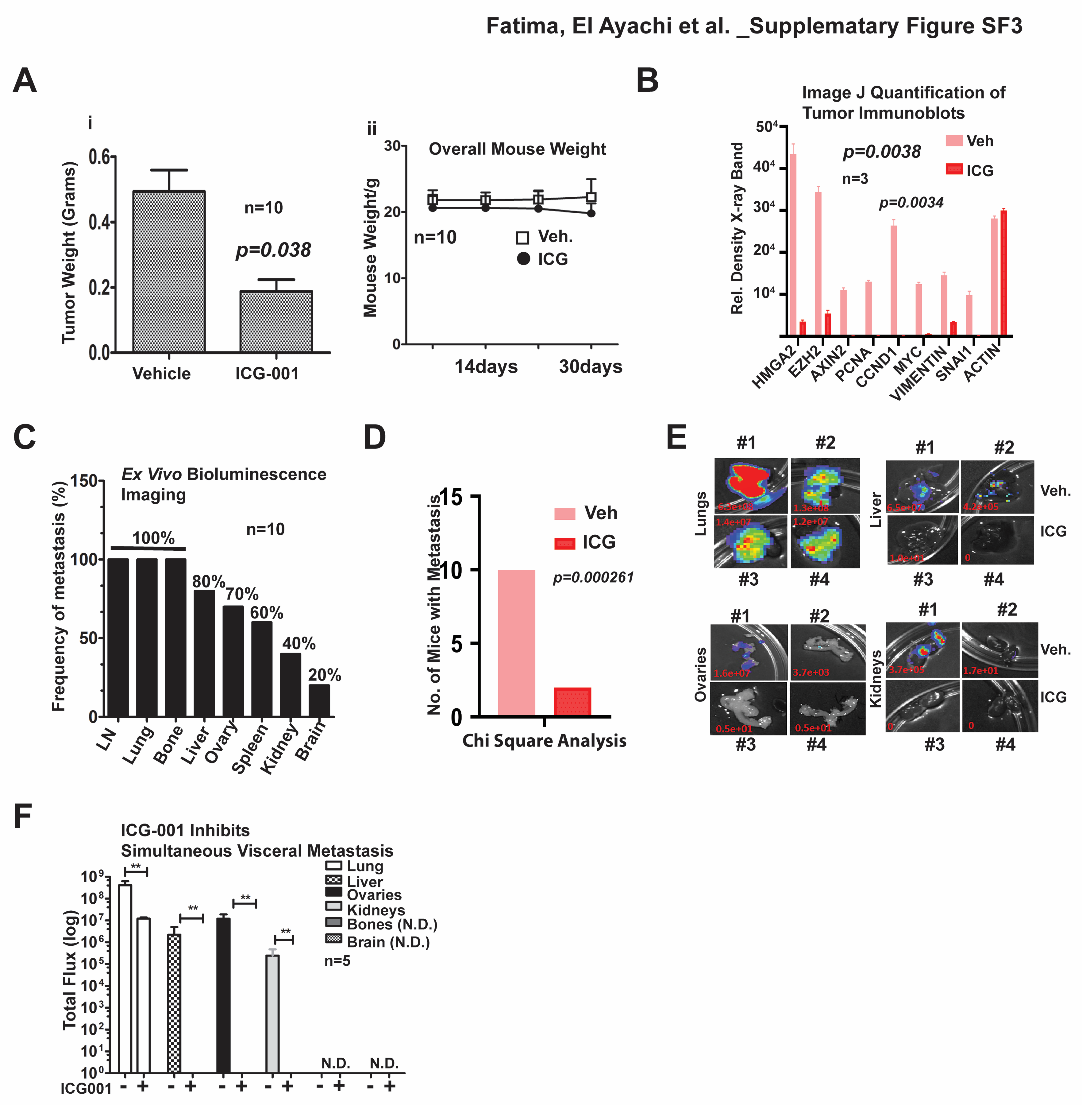


**Figure S3.** Wnt inhibition interferes with simultaneous visceral and bone metastasis in vivo in MDA-MB-231 cells. MDA-MB-231 stably transduced with lentivector-luciferase was used to track cells by bio-imaging after surgical transplantation into the mammary fat pad of NSG mice, beginning one week after initiation of ICG-001 therapy (200 mg/kg) given by IP every other day for two weeks. (**Ai**) Tumor weight tracked (n=10 mice) and overall weight from each mouse from the vehicle and ICG-001 treated groups (**Aii**). (**B**) ImageJ quantification of immunoblots from Figure 3, *P=value* was similar analyzed as in SF1C above. (**C**) Determination of the frequency of metastasis via ex vivo bioluminescence from lymph nodes (LN), lungs, bone, liver, ovary, spleen kidney and brains. (**D**) Number of mice with metastasis in vehicle vs. ICG-001 treated group. (**E**) Ex vivo bioluminescence images of the lungs, liver ovaries and kidneys, two mice each, from Veh and ICG-001 treated cohorts. (E) The number of mice with detectable metastasis is significantly decrease by exposure of ICG-001, *in vivo***.** (**F**) Ex vivo bioluminescence quantification for the lung, liver, ovary, kidney, bone and brain (N.D.) in the presence or absence of ICG-001demonstrates that metastasis to single organs was statistically significant decreased, in the presence of ICG-001. Statistics for *P=values*: (B) Similar analysis as in SF1C, (D) Chi Square analysis was used and (F) One-way analysis of variance using Newman-Keuls Multiple Comparison Test followed by Student’s *t-test,* two-tailed (***P<0.01)*.


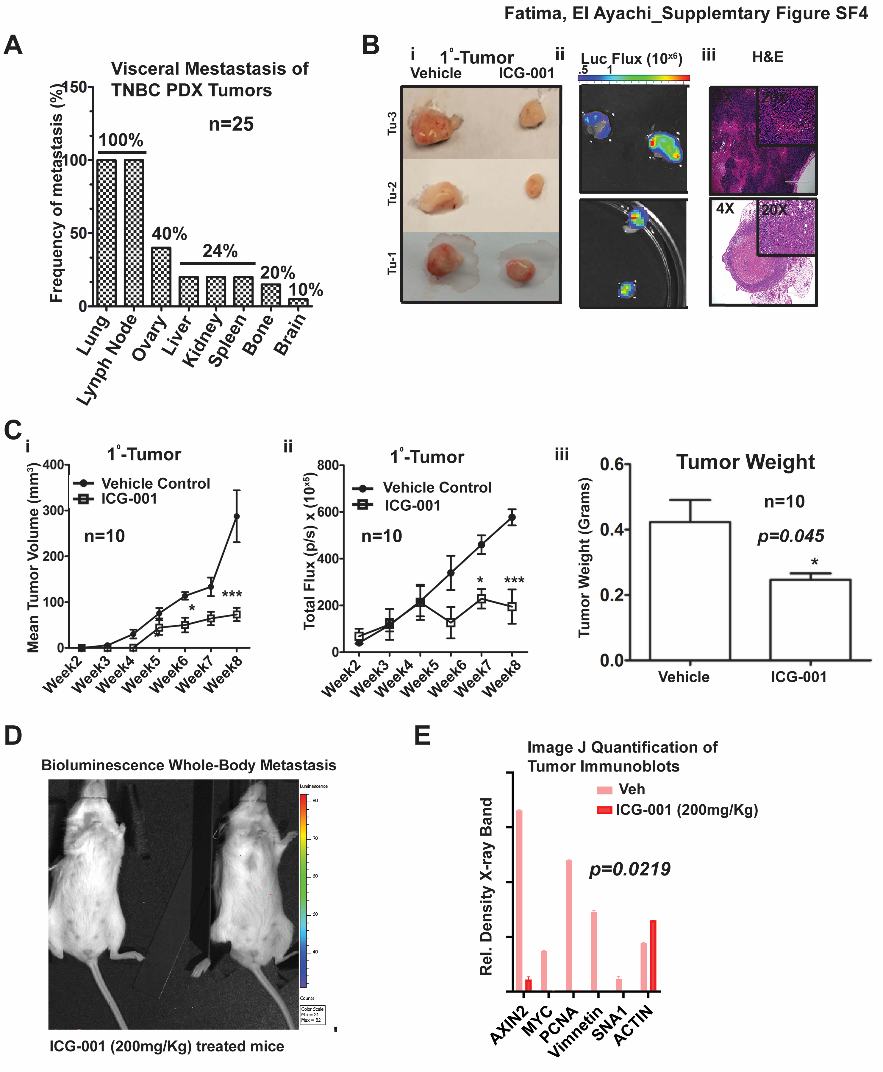


**Figure S4.** Wnt inhibition interferes with de novo whole body metastasis in highly chemoresistant TNBC PDX model. The TNBC PDX tumor HCI-10, which was stably transduced with lentivector-luciferase to track cells *in vivo*, was bilaterally transplanted into the mammary fat pad of NSG mice. Three weeks after transplantation, ICG-001 therapy was begun at a dose of either 100 or 200 mg/kg given every other day for two weeks. **A** Determination of the frequency of metastasis via ex vivo bioluminescence from lungs, lymph nodes (LN), ovary, liver, kidney, spleen bone, and brains. **B** Images from primary tumors (n=3) from Veh and ICG-001- treated cohorts (i), ex vivo bioluminescence images (ii) and H&E (iii). **C** primary tumor volume (i), total flux (p/s i.e., 10^5^) (ii) and tumor weight (iii) are all decreased in ICG-001-treated mice, n=10. *P-values* were generated by one-way ANOVA followed by pairwise Student’s *t-*tests (***P=>0.01*). **D** Whole-body luciferin images in two mice each from ICG-001 treated mice. **E** ImageJ quantification of immunoblots from Figure 4, that was similar analyzed as in SF1C above.


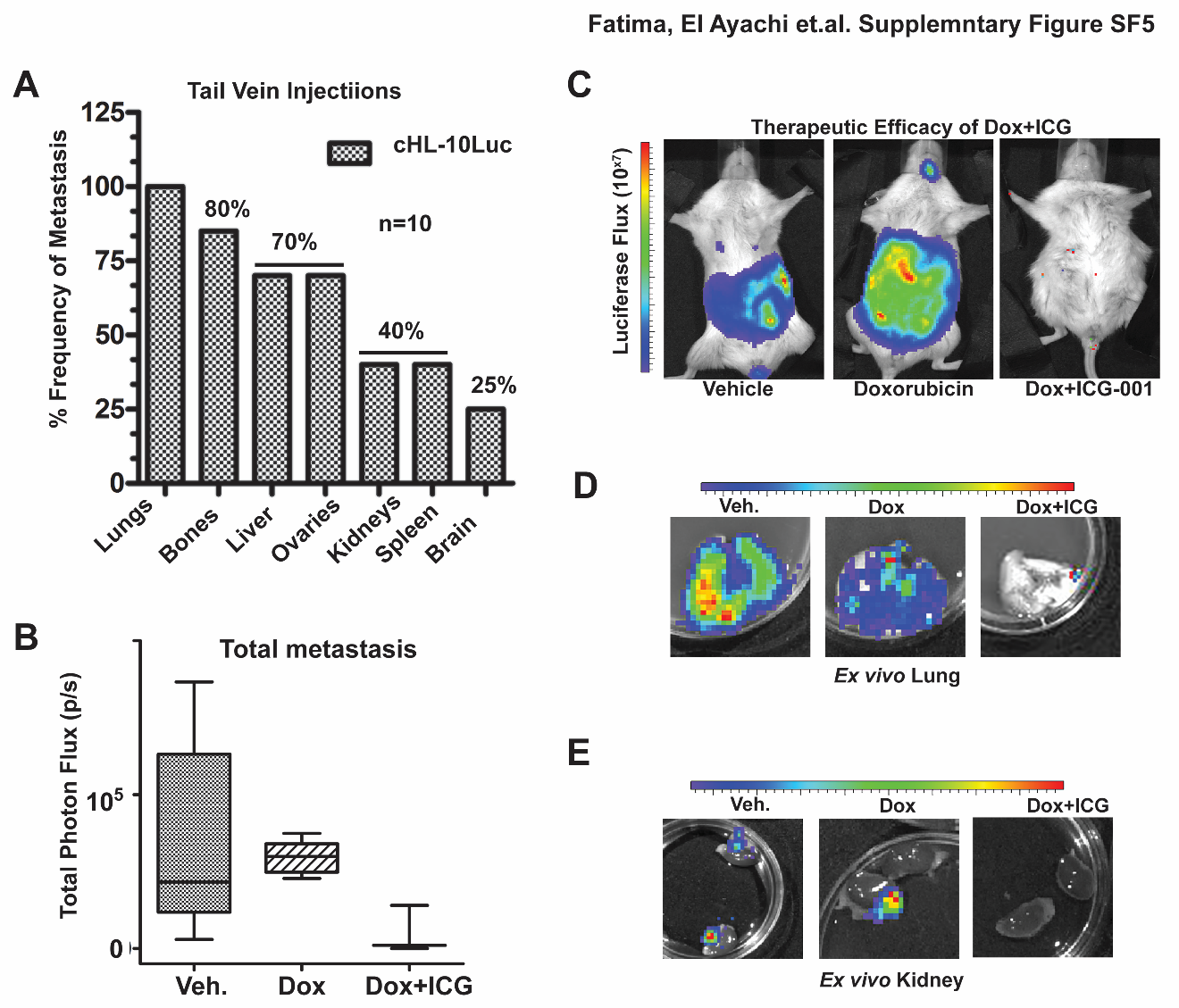


**Figure SF5.** ICG-001 sensitizes chemoresistant TNBC PDX tumor cells to doxorubicin, preventing metastasis. **A** cHCI-10 cells (1.25 × 10^6^) freshly isolated from primary PDX HCI-10Luc2 tumors were injected into the tail vein of NSG females to determine the frequency of metastasis to various organs. One day after tail vein injection, mice were treated with either DOX alone (1.4 mg/kg, IP) or DOX in combination with ICG-001 (50 mg/kg, IP) using the dosing schedules outlined in the materials and methods. Total Flux (p/s) was quantified by ex vivo bioluminescence imaging of the total metastasis for each cohort **B** by in vivo bioluminescence imaging in three mice **C** and by ex vivo bioluminescence imaging of the lungs **D** and kidneys **E**.

**Table 1.** Primer sequences used for qt-PCR.

| **Primer** | **Sequence** | **Orientation** |
| --- | --- | --- |
| *hAXIN2* | 5’-TCA AGT GCA AAC TTT CGC CAA CCG-3’ | S |
|  | 5’-TGG TGC AAA GAC ATA GCC AGA ACC-3’ | AS |
| *h-ACTIN* | 5’GGACTTCGAGCAAGAGATGG-3’ | S |
|  | 5’-AGC ACT GTG TTG GCG TAC AG-3’ | AS |
| *h-MYC* | 5’-TCT CCA CAC ATC AGC ACA ACT ACG-3’ | S |
|  | 5’-TGT GTT CGC CTC TTG ACA TTC TCC-3’ | AS |
| *hHMGA2* | 5’-GCC CCA GGA AGC AGC AGC AA-3’ | S |
|  | 5’-TCG AAC GTT GGC GCC CCC TA-3’ | AS |
| *hCCND1* | 5’-CAG AAG TGC GAA GAG GAG GTC-3’ | S |
|  | 5’-TCA TCT TAG AGG CCA CGA ACA T-3’ | AS |
| *hPCNA* | 5’-TGT AAA CCT GCA GAG CAT GGA CTC G-3’ | S |
|  | 5’- AAA TAC TAG CGC CAA GGT ATC CGC- 3’ | AS |
| *hGAPDH* | 5’-AAC AGC GAC ACC CAT CCT C-3’ | S |
|  | 5’-CATACCAGGAAATGAGCT TGACAA 3’ | AS |

Legend: Antisense (AS), Sense (S) and human (h).

Supplemental Materials and Methods

**Immunohistochemistry (IHC):** Tissues from breast tumors, adjacent normal breast and lung metastasis were fixed in 4% paraformaldehyde and embedded in paraffin. For in in vitro IF assays, cells were cultured in 8-well chamber slides (Thermo-Fisher Scientific, Waltham, MA, USA ) and fixed with 4% paraformaldehyde-PBS for 15 min, washed, incubated with blocking buffer (TBS pH 7.8, 3% BSA, 1% NGS, 1% Triton X-100, 0.01% NaAzide) and stained as follows. The following IHC primary antibody were used Hu anti mitochondria (ab92824). For all antibodies, a standard deparafinization and staining procedure was used as described in (1, 2). All IHC was performed manually without the use of automated immunostainers. Antigen retrieval was performed using a Decloaking Chamber (Biocare Medical, Concord, CA; 125ºC, 15 psi) in the presence of citrate buffer (pH 6.0). The total time slides were in the chamber was 45 min. Incubations with primary antibodies were performed at room temperature overnight in a humidified chamber. Normal goat serum was used for blocking. IHC kits anti-rabbit (K4003) or anti-mouse (K4001) secondary antibody with a DAB-CHROMOGEN (K3468) from DAKO were used for amplification and visualization of signal, respectively. Slides were counterstained with Hematoxylin QS (Vector Labs). IHC antibodies Cell Signaling (Beverly, MA, USA), CD31(#77699) and ABCAM (Cambridge, MA, USA), VEGFA (ab51745). Tissues known to contain each assessed antigen were used as positive controls.

**Animals:** All animal protocols and methods, including anesthesia, administration of drugs, and endpoints requiring euthanasia, were approved by the University of Tennessee Health Science Center (UTHSC) Animal Care and Use Committee (ACUC), consistent with the Guide for the Care and Use of Laboratory Animals, 8th edition as published by the National Academy of Sciences. All animals were maintained in a room with a 12 h light/dark cycle and provided food and water ad libitum.

**Cell extraction and immunoblotting blotting:** Protein extracts were prepared from cells exposed to the indicated ICG-001 IC50 concentration (maximum DMSO concentration 1%) and vehicle (1% DMSO). Treated cells were lysed with RIPA buffer (Cell Signaling, Beverly, MA, USA and immunoblotting was conducted as previously described 8, 20, 28. The following primary antibodies are from Cell Signaling: α-Actin (#3700,) Vimentin (#550513), non-phospho (Active) β-catenin (#8814), PCNA (#2586), HMGA2 (#5269), SLUG (C1967), EZH2 (5246S), Total PARP (#9532), Cleaved PARP (#5625), β-TUBULIN (#2128) and SNAIL (#4719). Antibodies from ABCAM (Cambridge, MA, USA) Axin2 (#ab32197) and from Santa Cruz Biotechnology, Inc (Dallas, TX, USA): Myc (#SC-764), CycinD1 (#SC10572) and Pan-β-catenin (#SC-7199). ImmunoPure peroxidase-conjugated secondary antibodies (Thermo-Fisher Scientific) were used according to manufacturer’s protocols. All experiments were conducted with both three biological and technical triplicates

**qPCR:** Isolation of total RNA was performed using TRIzol (Thermo-Fisher Scientific, Invitrogen) according to manufacturer’s protocol. RNA was treated with DNA-free™ DNA Removal Kit (Thermo-Fisher Scientific, Ambion) and converted to cDNA with Maxima First Strand cDNA Synthesis Kit (Thermo-Fisher Scientific, Fermentas) according to the manufacturer’s protocol. cDNA was subjected to quantitative PCR (qPCR) using the StepOnePlus (Thermo-Fisher Scientific, Applied Biosystems). qPCR was conducted in a final volume of 20 µL using Maxima SYBR Green/ROX qPCR Master Mix (Thermo-Fisher Scientific, Fermentas) according to the manufacturer’s protocols. Amplification conditions were: 95°C (5’), 40 cycles of 95°C (30s), 55°C (60s) and 72°C (60s). Primer pairs for each gene are provided in Supplemental Table S1. All experiments were conducted with both three biological and technical triplicates

**Generation of the drug combination index by isobole curves from WST-1 assay:** Both the non-adherent and adherent fractions of cHCI-10 Luc2 cells were trypsinized and then seeded at a density of 6,000 cells/well into flat-bottom well 96-well plates and allowed to plate overnight. ICG-001 (ApexBio, Houston, TX, USA) master stock (10 mM/100% DMSO, stored -20°C) was diluted immediately prior to use to 1 mM in DMSO as a working stock, which was diluted directly into growth medium. A doxorubicin (Sigma Aldrich, St. Louis, MO, USA) master stock (5 mM/100% DMSO, stored -20°C) and diluted directly into growth medium. The dose range for ICG-001 was 0.1 μM to 30 μM (0.1 μM, 1 μM, 5 μM, 10 μM, 15 μM, 20 μM and 30 μM) and the dose range for doxorubicin was 0.02 μM to 30 μM (0.02 μM, 0.04μM, 0.2 μM, 1 μM, 5 μM, 10 μM, 20μM, 30 μM). Each drug was added alone or in combination with the other drug to cells (n=3 technical replicates/condition) along with the appropriate vehicle controls (diluted DMSO alone). Drug(s) were not re-added or the medium changed over the course of the experiment (48 h). At 48h, cells were incubated with WST-1 reagent (Sigma-Millipore, St. Louis, MO, USA) and read at 440 nm on a spectrophotometer. Combination indices were calculated for the 48-h time point per (1).

**Generation of Luciferase2 (Luc2)-labeled HCI PDX models:** Cryopreserved tumor fragments derived from previously characterized patient-derived xenograft (PDX) breast tumors (Caucasian, EA patients) were generously provided by the laboratory of Dr. Alana Welm and the Huntsman Cancer Institute (Salt Lake City, UT, USA ) tissue resource and application core (MDA-MB-231, HCI-1, HCI-2, HCI-3, HCI-9 and HCI-10). Upon receipt at UTHSC, tumor fragments were thawed, washed and re-implanted into NOD/SCID/ILR2/GAMMA (NSGγ) immunocompromised female recipients by orthotopic implantation into the right inguinal mammary fat pad(s). Individual PDX tumor lines were then serially re-passaged by transplantation of tumor fragments (2 x 2 mm) into the cleared mammary fat pads of NSG females at 4- 8 weeks of age. Protein extracts were prepared from four triple negative breast cancer (TNBC) lines (HCI-1, HCI-2, HCI-9 and HCI-10) and one Luminal B (HER2-) line, HCI-3, using the protocols described in (5). To facilitate bio-imaging, the metastatic TNBC lines HCI-2 and HCI-10 PDX tumor lines were labeled with firefly luciferase2 (derived from pGL4, Promega Corporation, Madison, WI, USA). Parental PDX tumors were resected from anesthetized animals, necrotic areas were removed and healthy tumor tissue was chopped to a fine paste and then digested with collagenase III (Worthington Biochemical Corporation, Lakewood, NJ, USA) and hyaluronidase (Sigma-Aldrich, St. Louis, MO, USA ) in DMEM/F12 + 2% FBS using methods outlined in (ref #4). Pelleted large organoids were then digested with dispase II (Roche) to generate a population of single cells/small organoids. Following a series of washes, isolated tumor epithelial cells were plated at a density of 250,000 cells/well into 6- well ultra-low adhesion dishes containing breast stem cell media (DMEM/F12, 1× B27, 20 ng/mL human EGF, 20 ng/mL bFGF and 4 μg/mL heparin). The next day, cells were transduced overnight in the presence of 8 μg/mL polybrene with a m.o.i. of 10 of lentivirus that expressed luciferase2 and puromycin (Lentivirus-EF1alpha-Luciferase-IRES-puro, pEILP, using the pHIV-Luciferase backbone, AddGene (Watertown, MA, USA, plasmid #21375). The next day, the cells were washed with HBSS+ to remove virus and were replated into fresh stem cell media. Forty-eight hours after transduction, cells were selected for luciferase expression by the addition of 1 µg/mL of puromycin for 3-6 days. Transduced cells were centrifuged at low speed to enrich for live cells and the pellet resuspended in a 1:1 mixture of HBSS+/growth-factor reduced Matrigel and transplanted into the cleared mammary fat pads of NSG female mice. After initial tumor formation, which was monitored by bio-imaging (Xenogen IVIS Lumina, Perkin Elmer Akron, OH, USA), the subsequent generation of HCI-2Luc2 or HCI-10-Luc2 TNBC tumors were generated using standard tumor fragment repassaging into NSG recipients. Cell lines were generated by digestion of the second generation of HCI-2Luc2 or HCI-10Luc2 PDX tumors using protocols outlined in (2) and cultured in M87 complete medium in standard tissue culture dishes. Protein extracts were prepared from four triple negative breast cancer (TNBC) lines passaged in NSGγ mice (HCI-1, HCI-2, HCI-9 and HCI-10) using protocols described in (2).

References:

1. Tallarida, R.J. An overview of drug combination analysis with isobolograms. *J. Pharmacol. Exp. Ther.* **2006**, *319*, 1–7.

2. El Ayachi, I.; Fatima, I.; Wend, P.; Alva-Ornelas, J.A.; Runke, S.; Kuenzinger, W.L.; Silva, W.; Gray, J.K.; Lehr, S.; et al. The WNT10B network is associated with survival and metastases in chemoresistant triple-negative breast cancer. *Cancer Res.* **2019**, *79*, 982–993, doi: 10.1158/0008-5472.

| 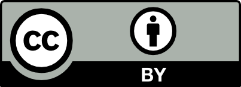 | © 2019 by the authors. Licensee MDPI, Basel, Switzerland. This article is an open access article distributed under the terms and conditions of the Creative Commons Attribution (CC BY) license (http://creativecommons.org/licenses/by/4.0/). |
| --- | --- |
