# Supplementary figures and images for: Simultaneous Multi-Organ Metastases from Chemo-Resistant Triple-Negative Breast Cancer Are Prevented by Interfering with WNT-Signaling
Source: Cancers (Basel). 2019 Dec 17;11(12):2039. doi: 10.3390/cancers11122039 (PMC6966654; doi:10.3390/cancers11122039)

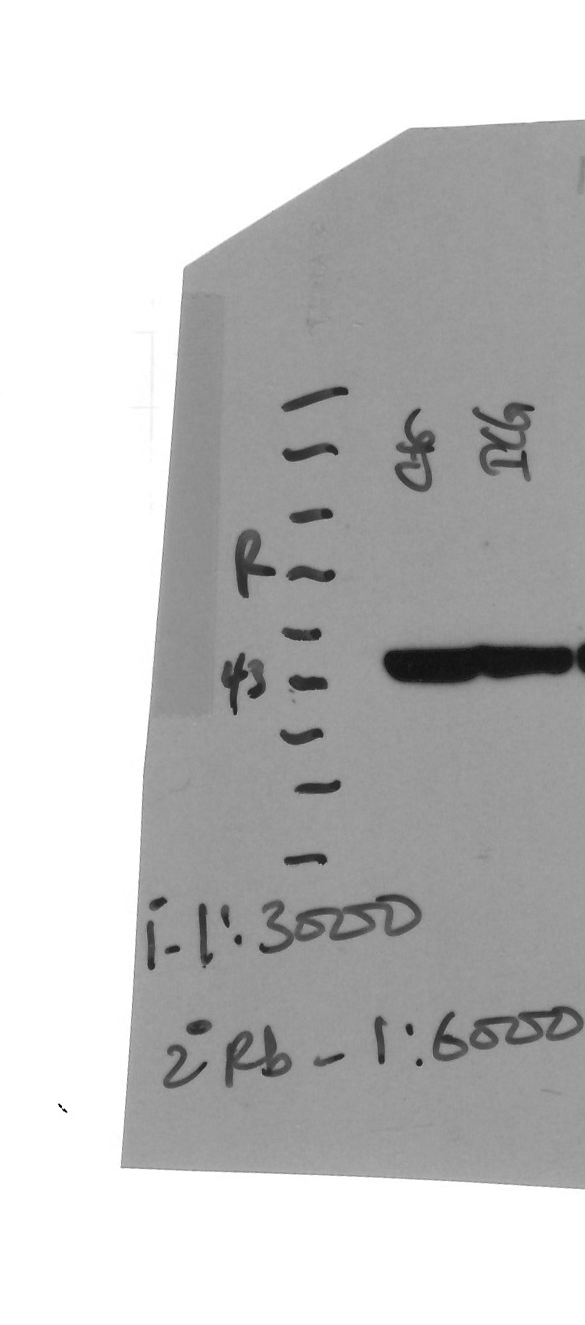

Supplement: Supplementary file 1 [file cancers-11-02039-s001.zip › cancers-631322-suppl-final2/Fig 1 MDA-157/ACTIN_ MDA157.tif]

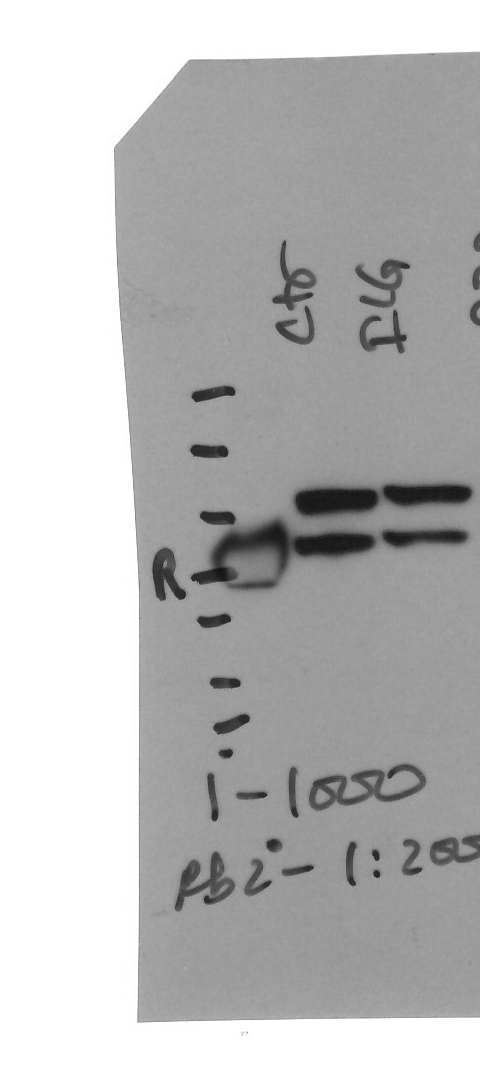

Supplement: Supplementary file 1 [file cancers-11-02039-s001.zip › cancers-631322-suppl-final2/Fig 1 MDA-157/AXIN2_ MDA157.tif]

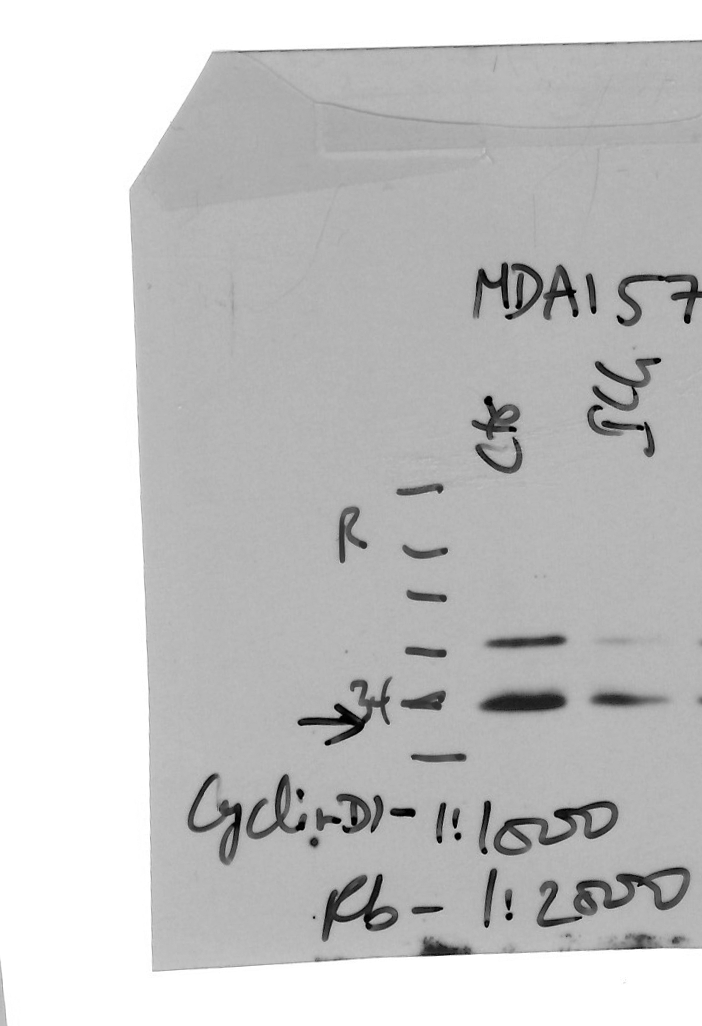

Supplement: Supplementary file 1 [file cancers-11-02039-s001.zip › cancers-631322-suppl-final2/Fig 1 MDA-157/Cyclin D1_ MDA157.tif]

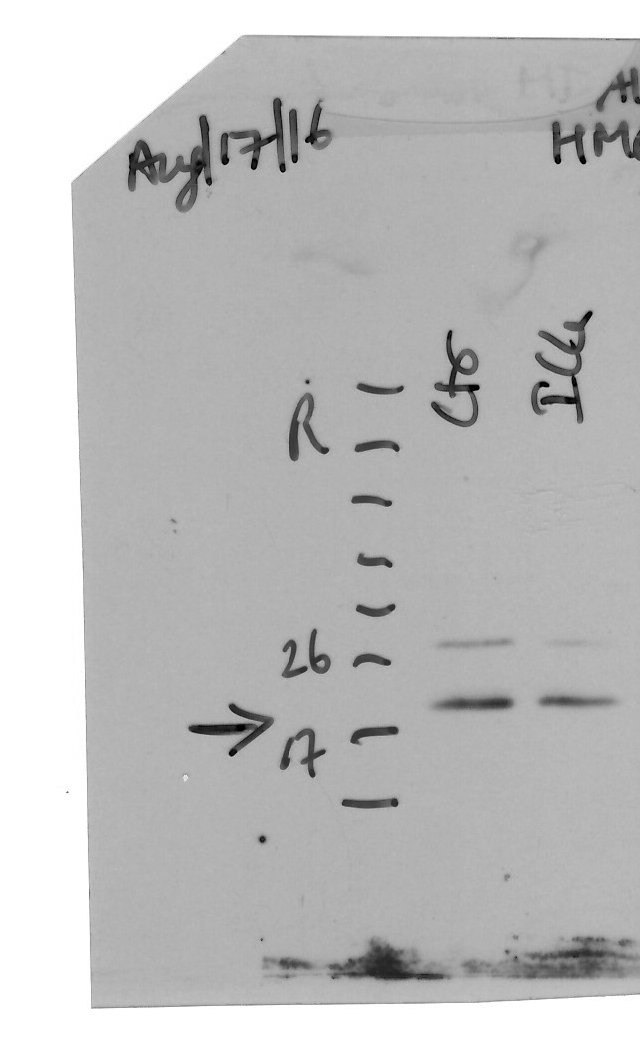

Supplement: Supplementary file 1 [file cancers-11-02039-s001.zip › cancers-631322-suppl-final2/Fig 1 MDA-157/HMGA2_ MDA157.tif]

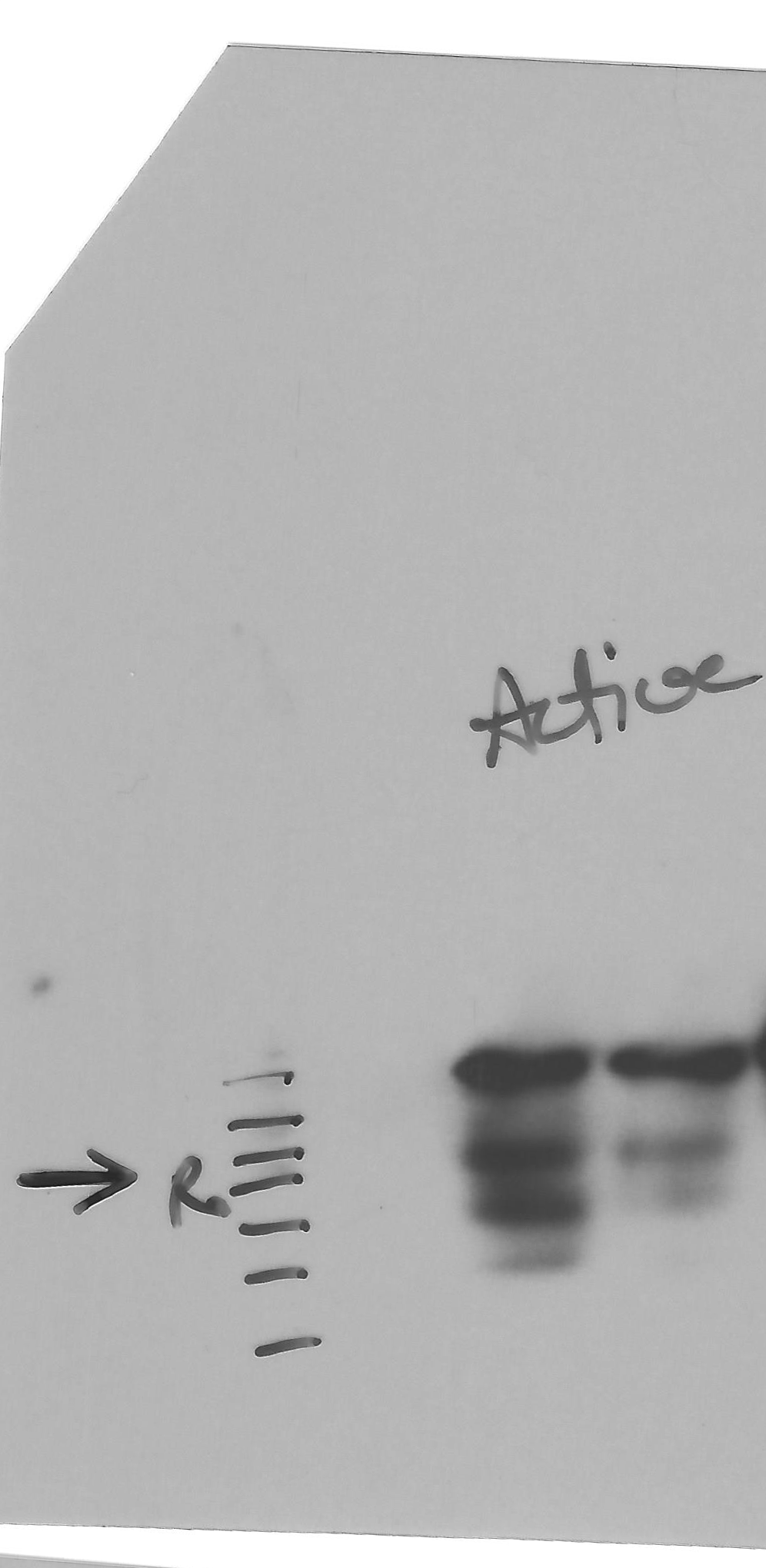

Supplement: Supplementary file 1 [file cancers-11-02039-s001.zip › cancers-631322-suppl-final2/Fig 1 MDA-157/Non phospho beta catenin_ MDA157.tif]

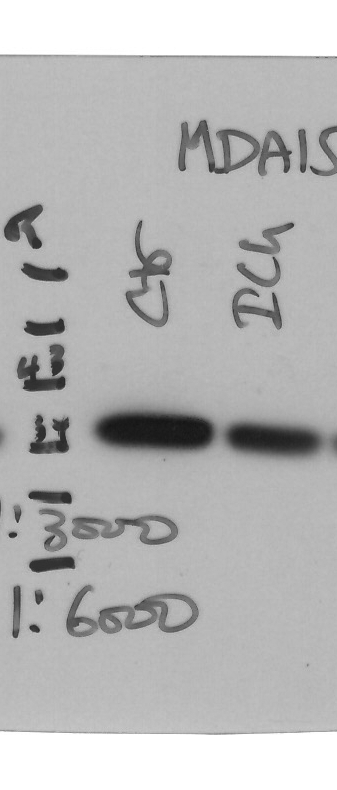

Supplement: Supplementary file 1 [file cancers-11-02039-s001.zip › cancers-631322-suppl-final2/Fig 1 MDA-157/PCNA_ MDA157.tif]

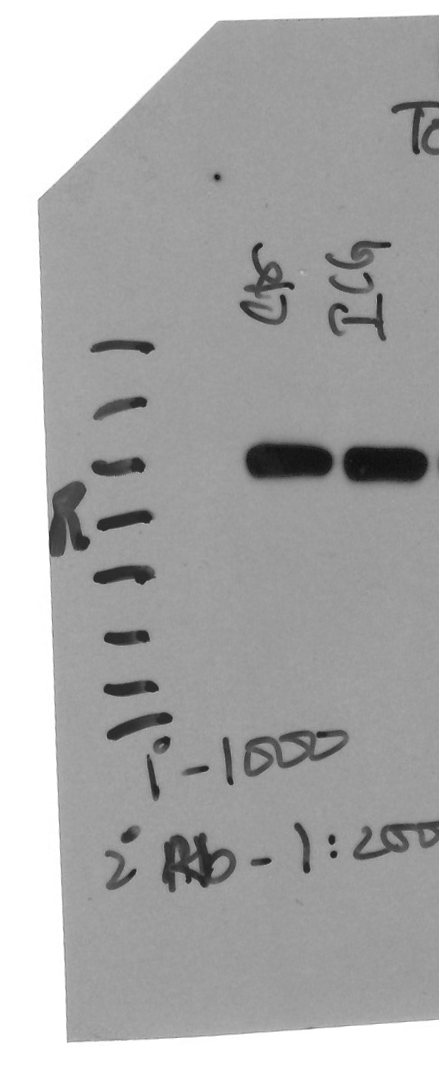

Supplement: Supplementary file 1 [file cancers-11-02039-s001.zip › cancers-631322-suppl-final2/Fig 1 MDA-157/total beta catenin_ MDA157.tif]

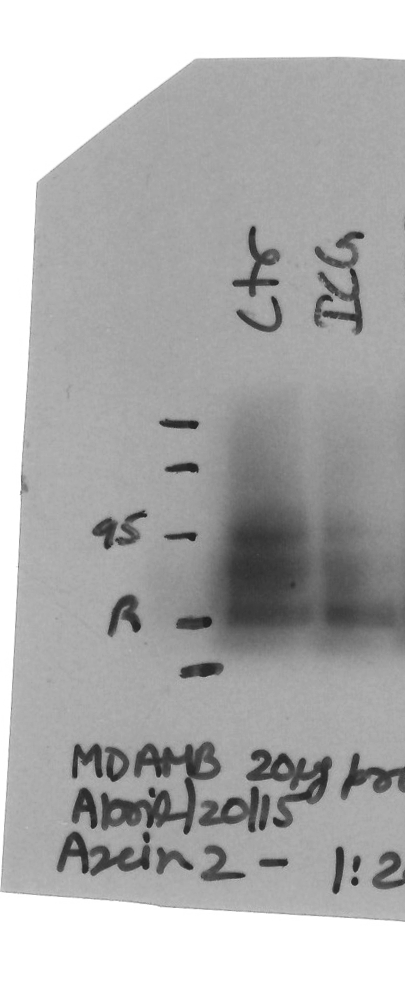

Supplement: Supplementary file 1 [file cancers-11-02039-s001.zip › cancers-631322-suppl-final2/Fig 1 MDA-231/AXIN2_ MDA231.tif]

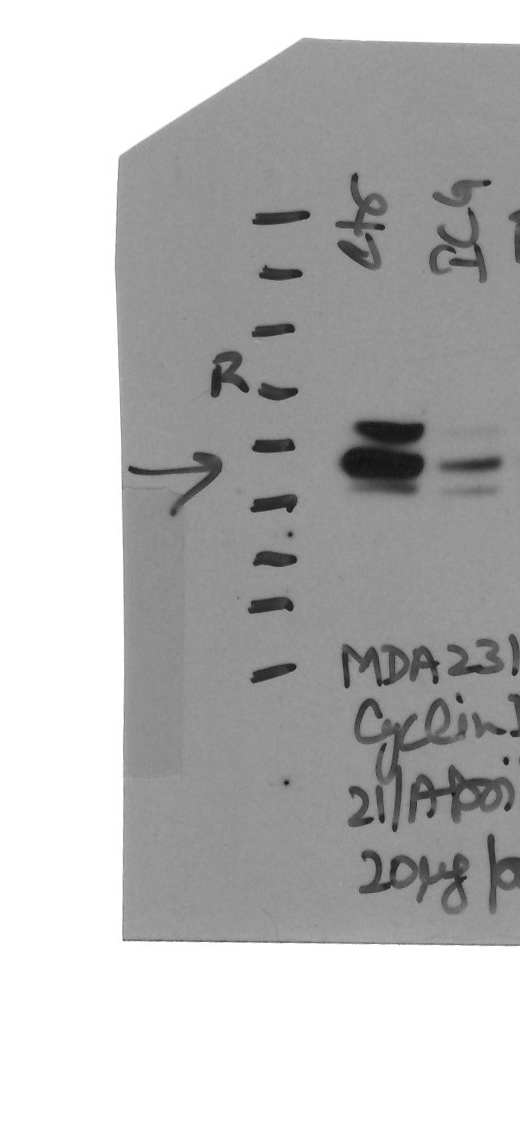

Supplement: Supplementary file 1 [file cancers-11-02039-s001.zip › cancers-631322-suppl-final2/Fig 1 MDA-231/CCND1_ MDA231.tif]

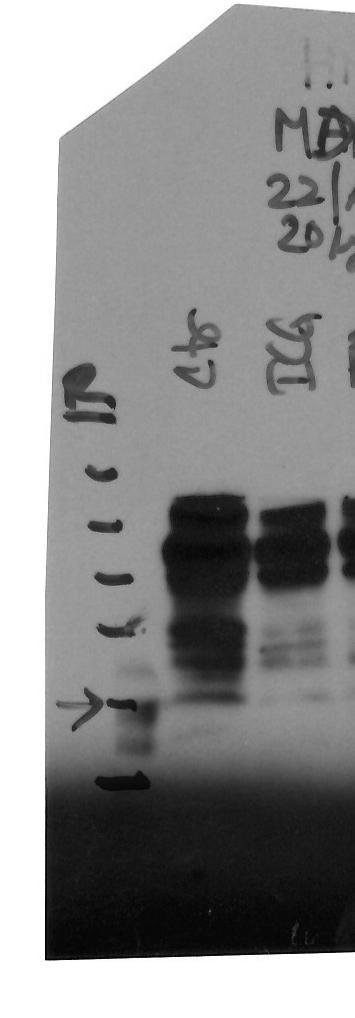

Supplement: Supplementary file 1 [file cancers-11-02039-s001.zip › cancers-631322-suppl-final2/Fig 1 MDA-231/HMGA2_ MDA231.tif]

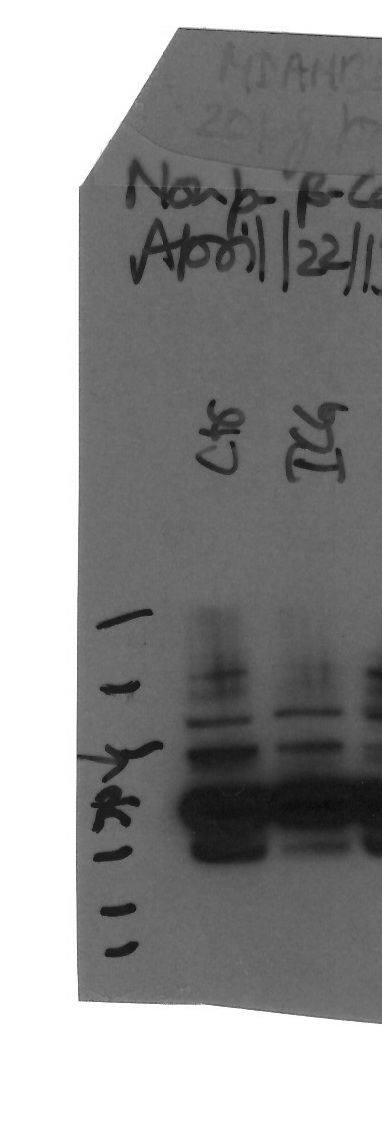

Supplement: Supplementary file 1 [file cancers-11-02039-s001.zip › cancers-631322-suppl-final2/Fig 1 MDA-231/Non phospho beta catenin_ MDA231.tif]

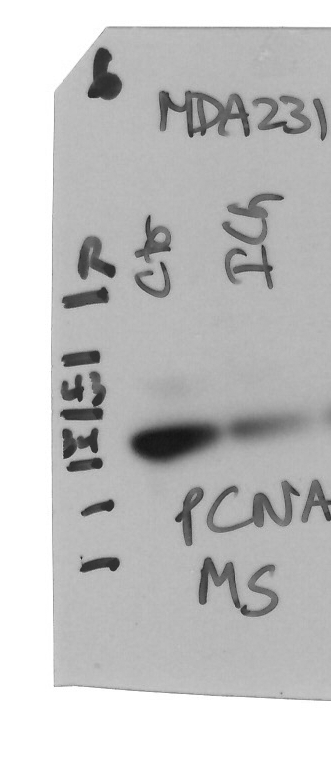

Supplement: Supplementary file 1 [file cancers-11-02039-s001.zip › cancers-631322-suppl-final2/Fig 1 MDA-231/PCNA_ MDA231.tif]

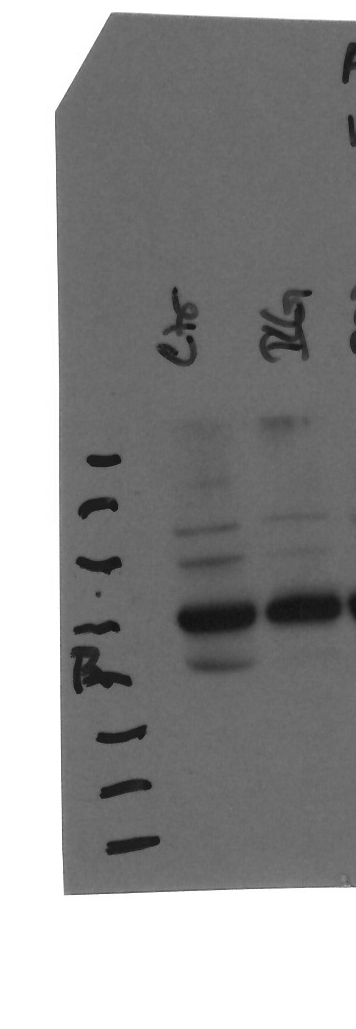

Supplement: Supplementary file 1 [file cancers-11-02039-s001.zip › cancers-631322-suppl-final2/Fig 1 MDA-231/total beta catenin_ MDA231.tif]

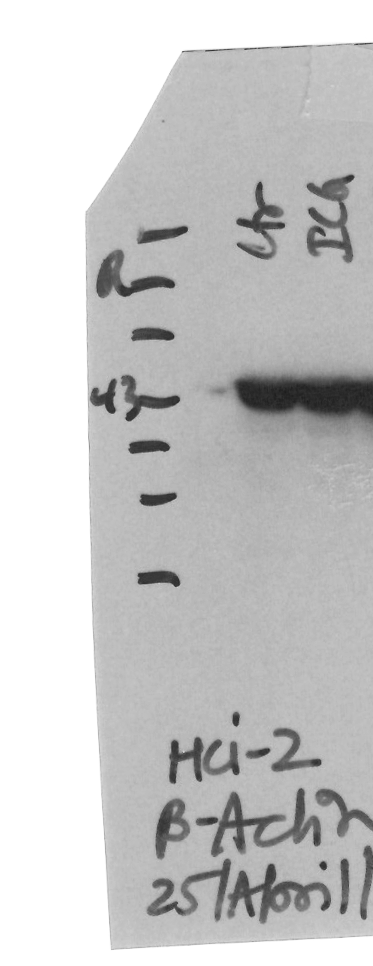

Supplement: Supplementary file 1 [file cancers-11-02039-s001.zip › cancers-631322-suppl-final2/Fig1 HCI-2/ACTIN_HCI-2.tif]

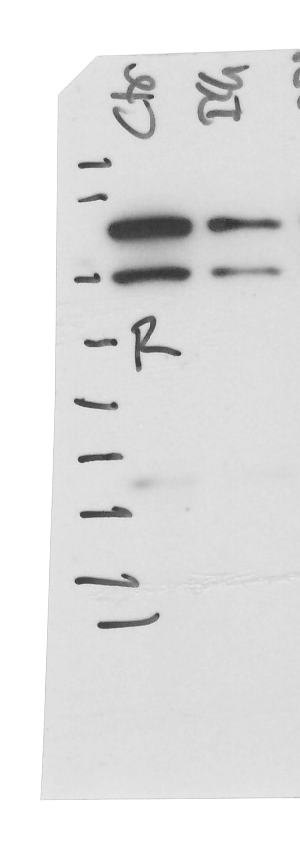

Supplement: Supplementary file 1 [file cancers-11-02039-s001.zip › cancers-631322-suppl-final2/Fig1 HCI-2/AXIN2_HCI-2.tif]

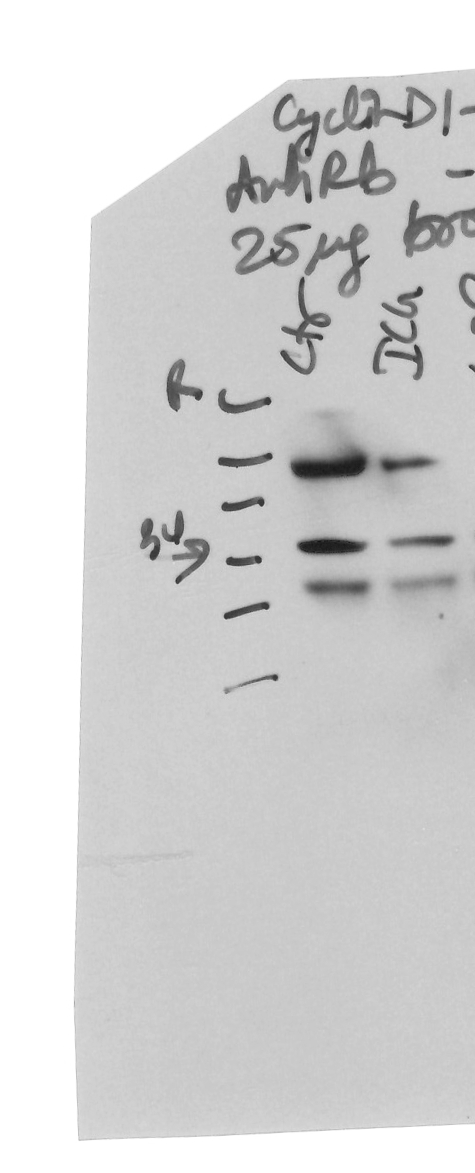

Supplement: Supplementary file 1 [file cancers-11-02039-s001.zip › cancers-631322-suppl-final2/Fig1 HCI-2/CyclinD1_HCI-2.tif]

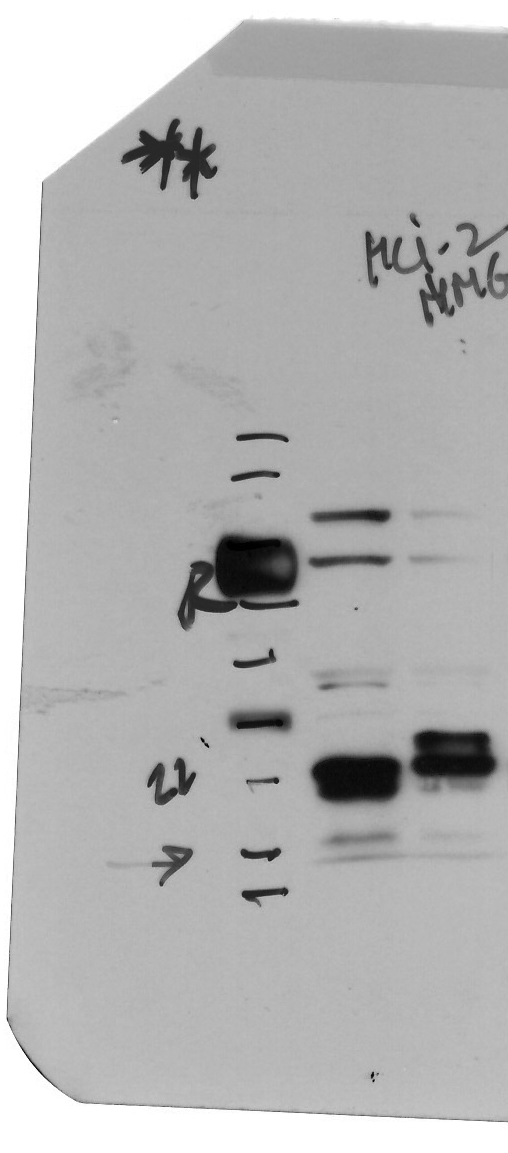

Supplement: Supplementary file 1 [file cancers-11-02039-s001.zip › cancers-631322-suppl-final2/Fig1 HCI-2/HMGA2_HCI-2.tif]

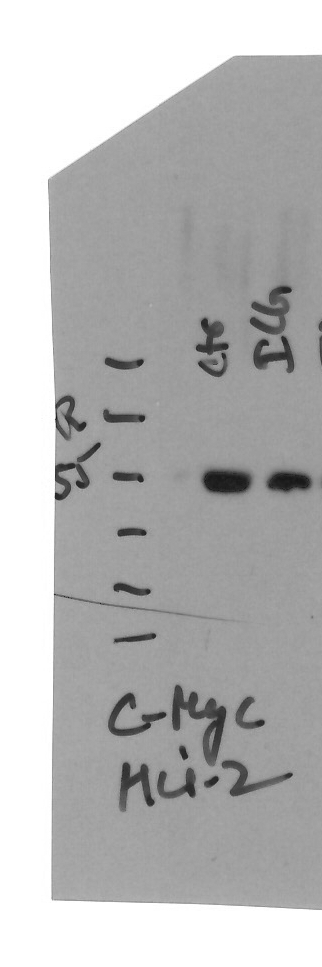

Supplement: Supplementary file 1 [file cancers-11-02039-s001.zip › cancers-631322-suppl-final2/Fig1 HCI-2/Myc_HCI-2.tif]

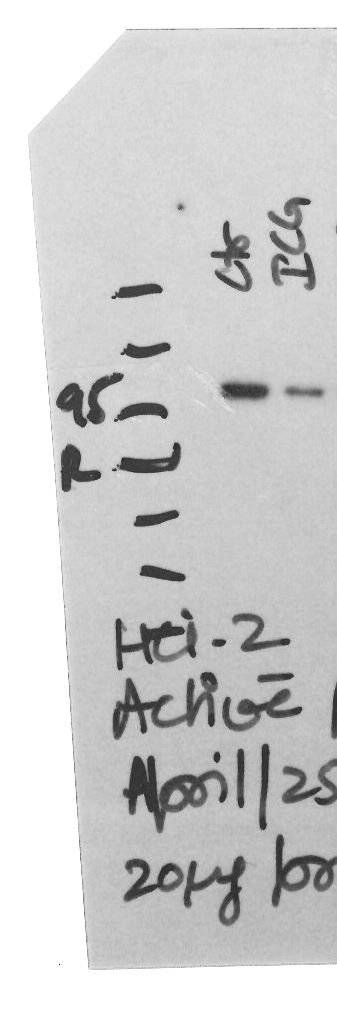

Supplement: Supplementary file 1 [file cancers-11-02039-s001.zip › cancers-631322-suppl-final2/Fig1 HCI-2/non phopho beta catenin_HCI-2.tif]

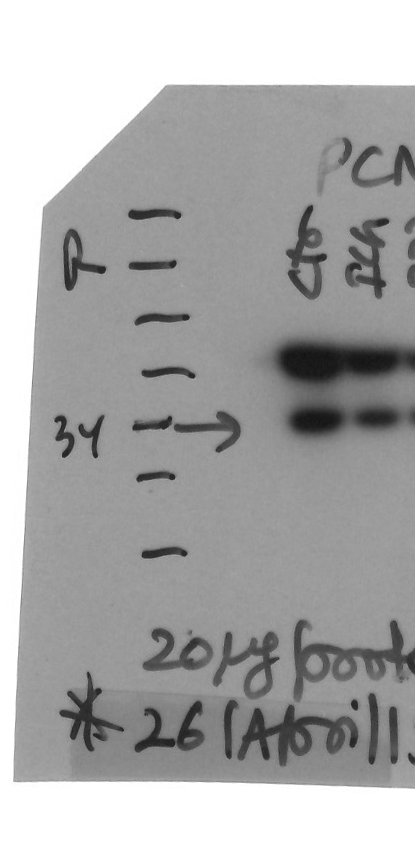

Supplement: Supplementary file 1 [file cancers-11-02039-s001.zip › cancers-631322-suppl-final2/Fig1 HCI-2/PCNA_HCI-2.tif]

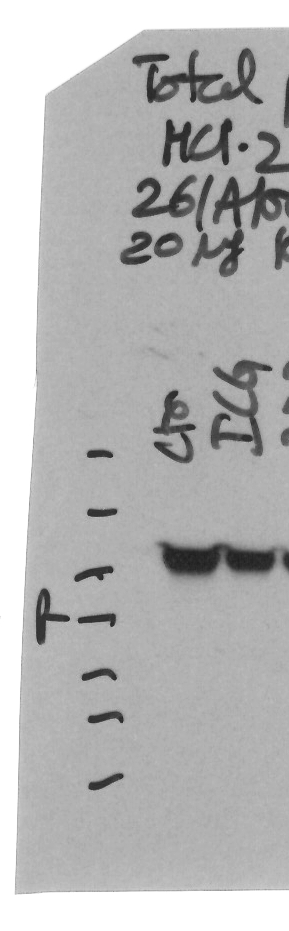

Supplement: Supplementary file 1 [file cancers-11-02039-s001.zip › cancers-631322-suppl-final2/Fig1 HCI-2/total beta catenin_HCI-2.tif]

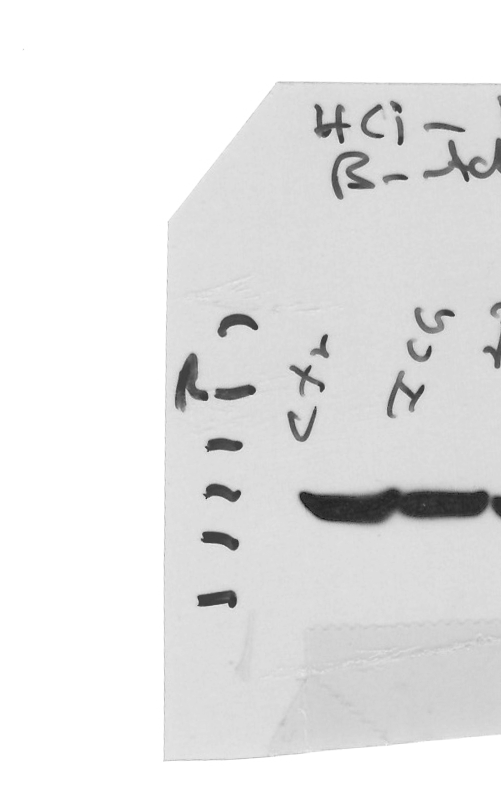

Supplement: Supplementary file 1 [file cancers-11-02039-s001.zip › cancers-631322-suppl-final2/Fig1 HCI10/ACTIN_HCI-10.tif]

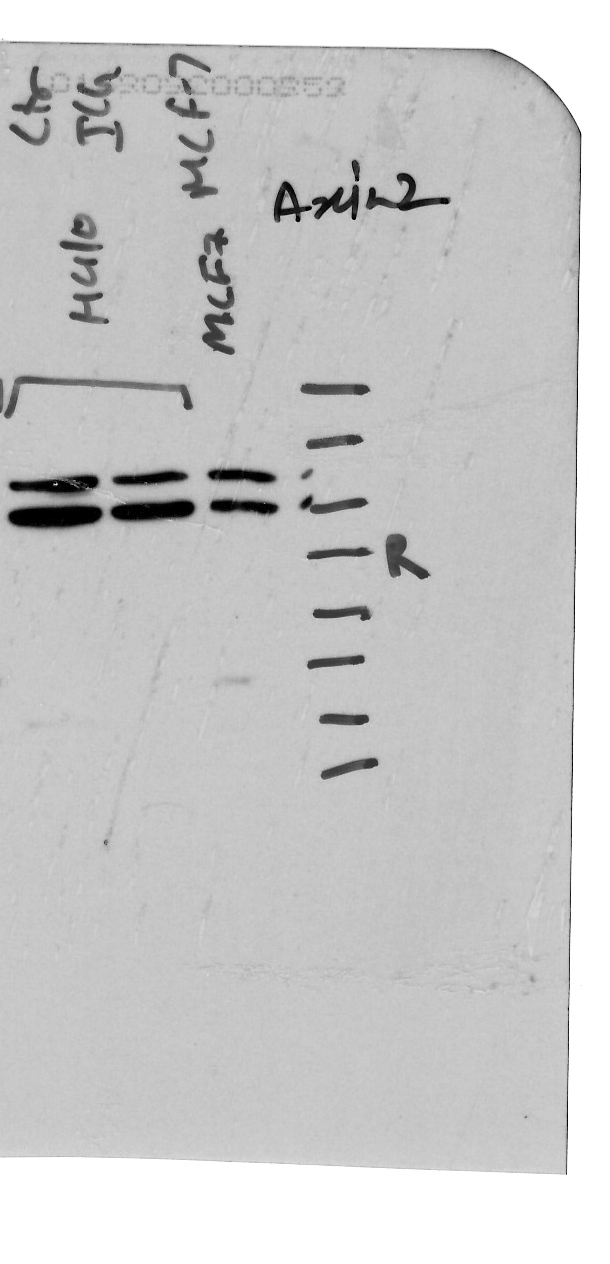

Supplement: Supplementary file 1 [file cancers-11-02039-s001.zip › cancers-631322-suppl-final2/Fig1 HCI10/AXIN2_HCI-10.tif]

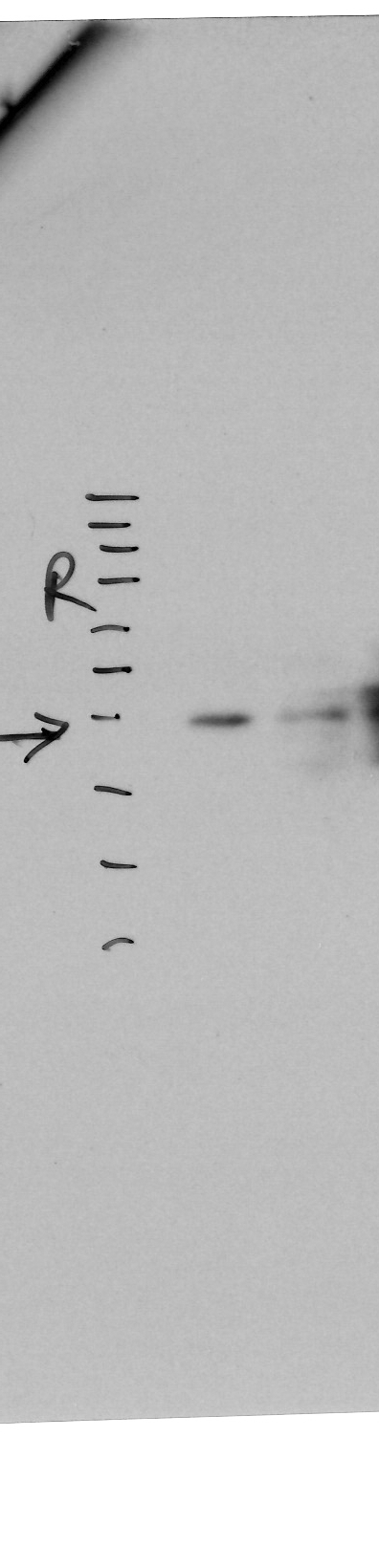

Supplement: Supplementary file 1 [file cancers-11-02039-s001.zip › cancers-631322-suppl-final2/Fig1 HCI10/CyclinD1_HCI-10.tif]

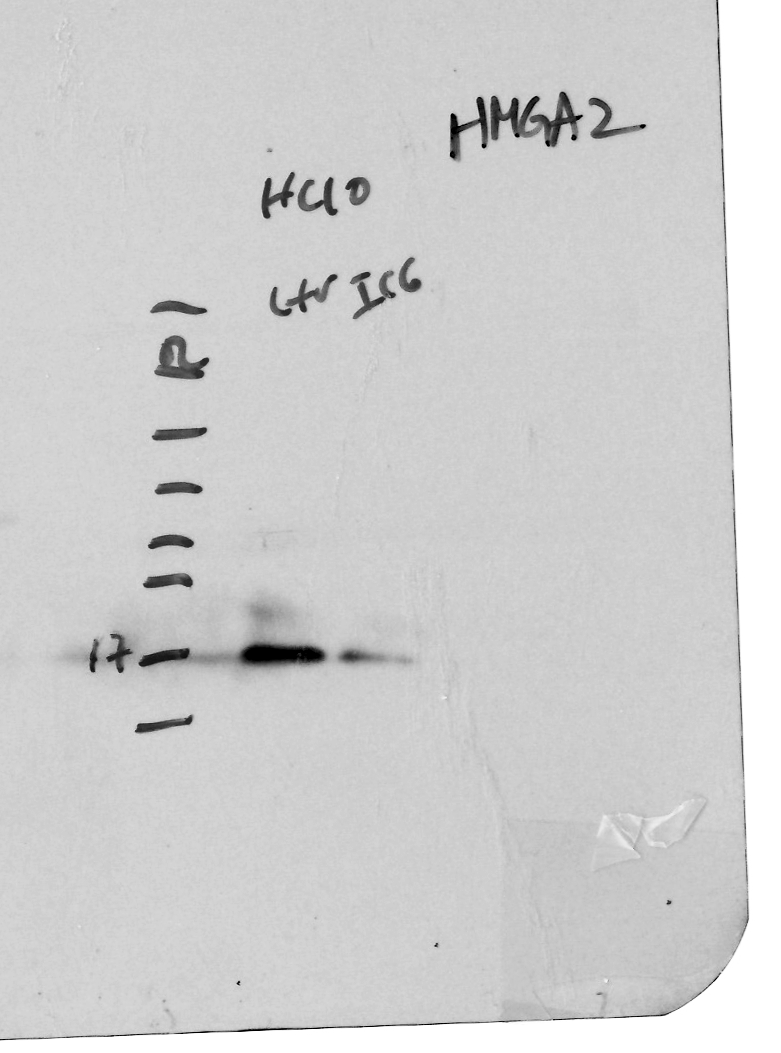

Supplement: Supplementary file 1 [file cancers-11-02039-s001.zip › cancers-631322-suppl-final2/Fig1 HCI10/HMGA2_HCI-10.tif]

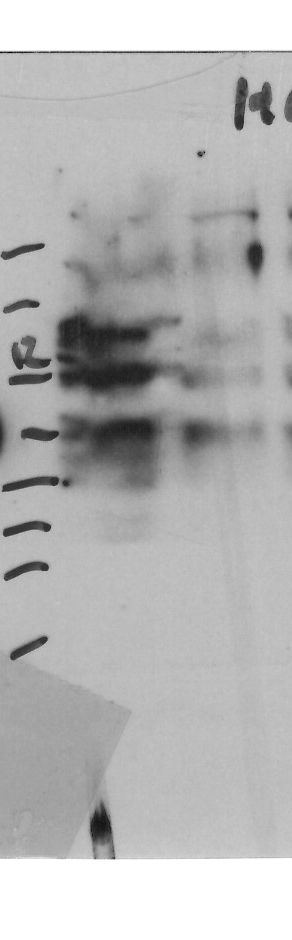

Supplement: Supplementary file 1 [file cancers-11-02039-s001.zip › cancers-631322-suppl-final2/Fig1 HCI10/Myc_HCI-10.tif]

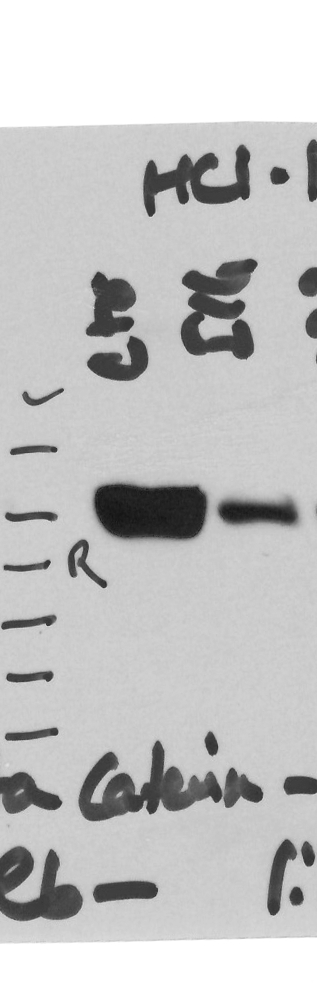

Supplement: Supplementary file 1 [file cancers-11-02039-s001.zip › cancers-631322-suppl-final2/Fig1 HCI10/non phopho beta catenin_HCI-10.tif]

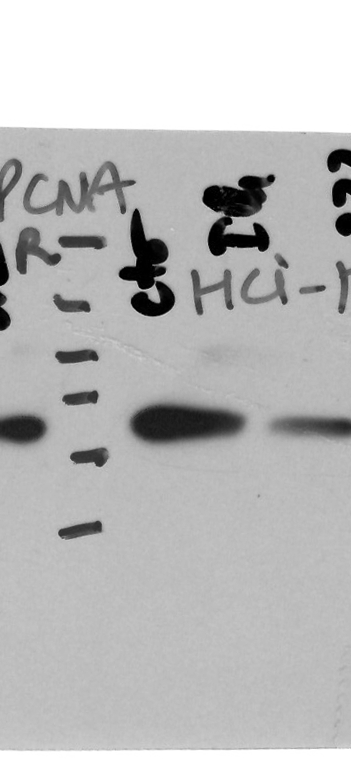

Supplement: Supplementary file 1 [file cancers-11-02039-s001.zip › cancers-631322-suppl-final2/Fig1 HCI10/PCNA_HCI-10.tif]

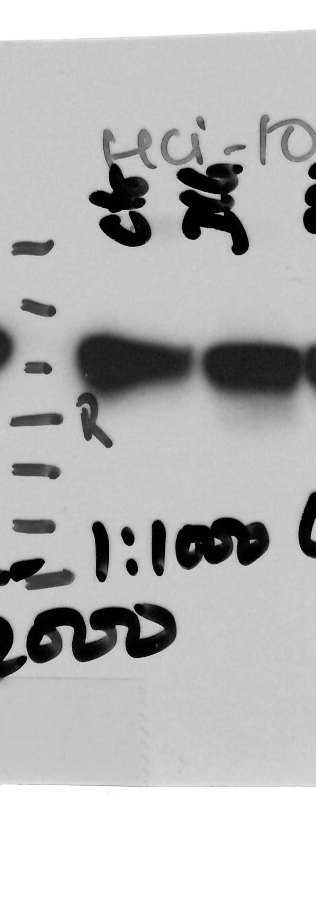

Supplement: Supplementary file 1 [file cancers-11-02039-s001.zip › cancers-631322-suppl-final2/Fig1 HCI10/total beta catenin_HCI-10.tif]

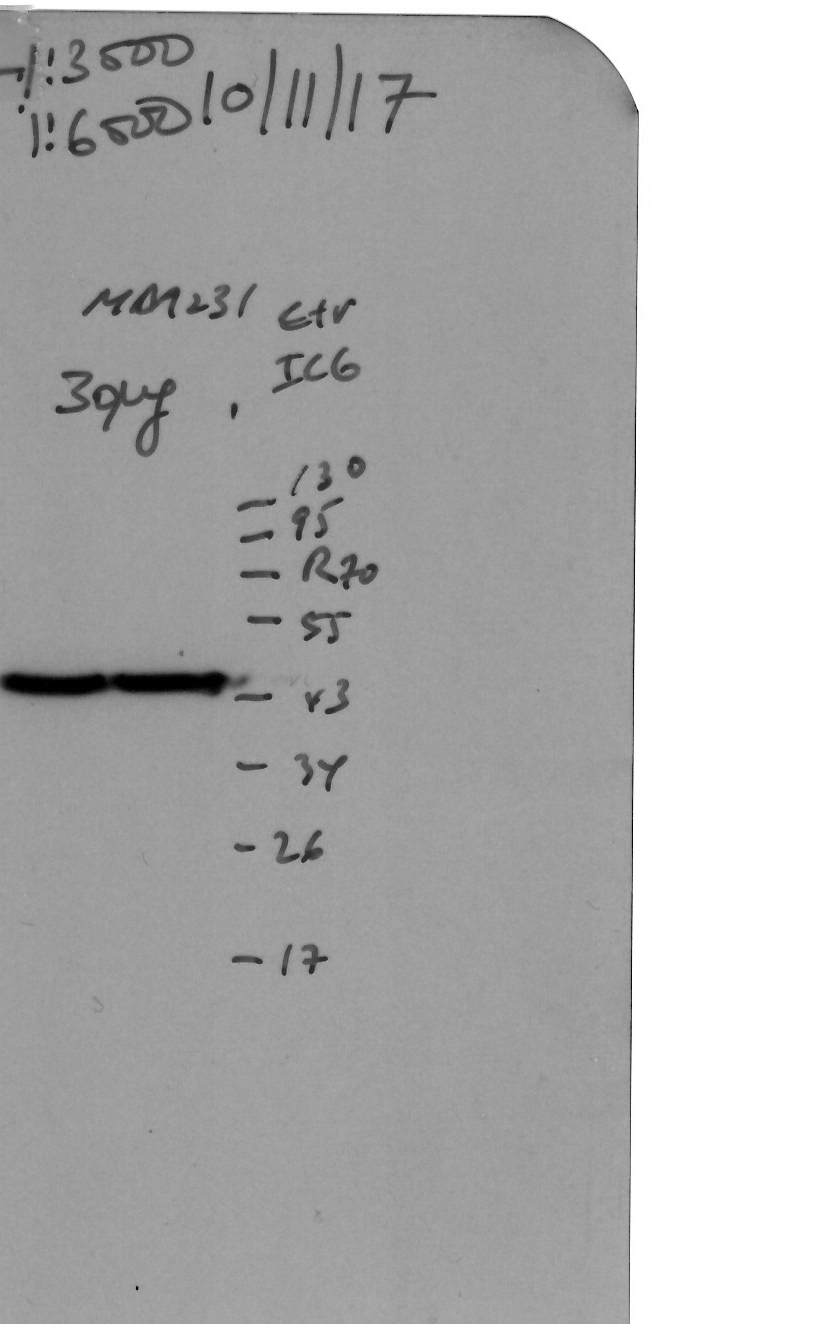

Supplement: Supplementary file 1 [file cancers-11-02039-s001.zip › cancers-631322-suppl-final2/Fig3_IB/ACTIN MDAMD 231 In vivo.tif]

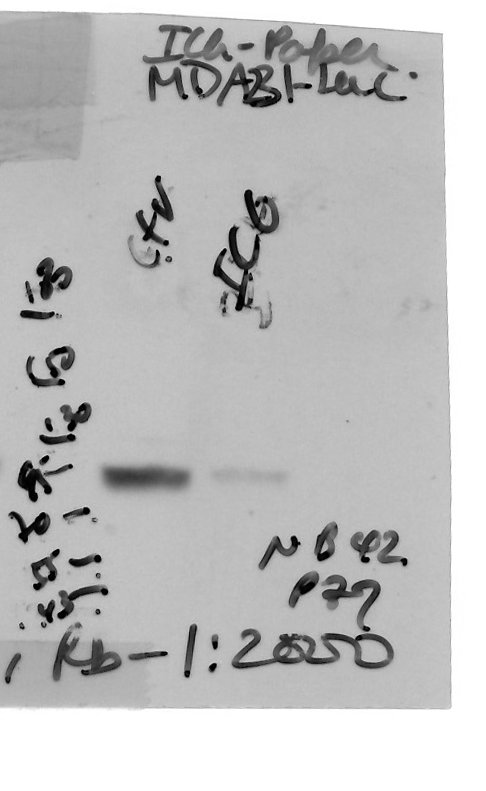

Supplement: Supplementary file 1 [file cancers-11-02039-s001.zip › cancers-631322-suppl-final2/Fig3_IB/Axin2 MDAMD 231 In vivo.tif]

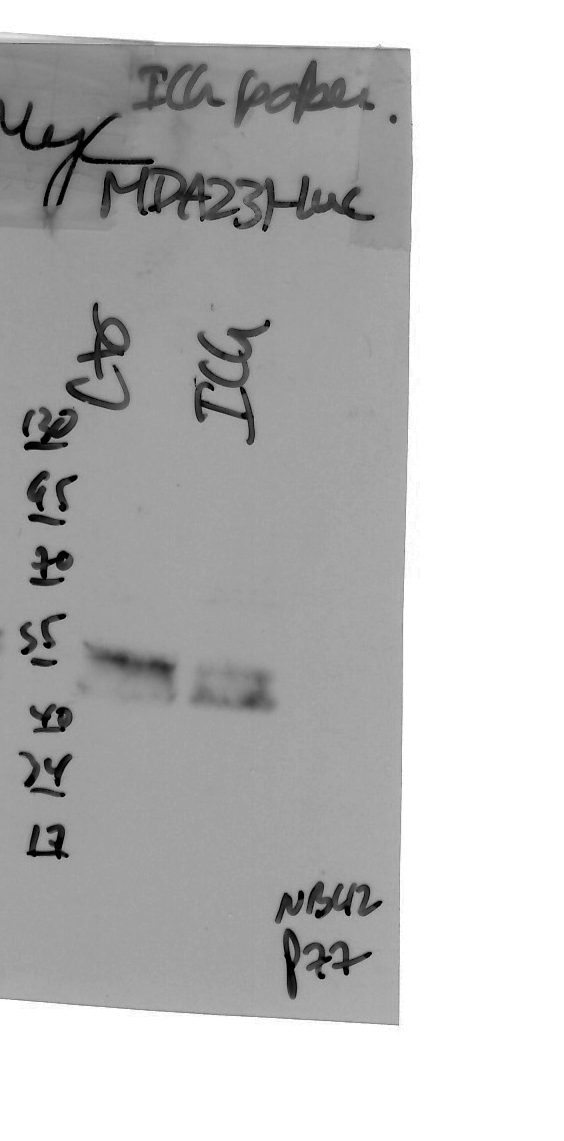

Supplement: Supplementary file 1 [file cancers-11-02039-s001.zip › cancers-631322-suppl-final2/Fig3_IB/cMyc_MDAMD 231 In vivo.tif]

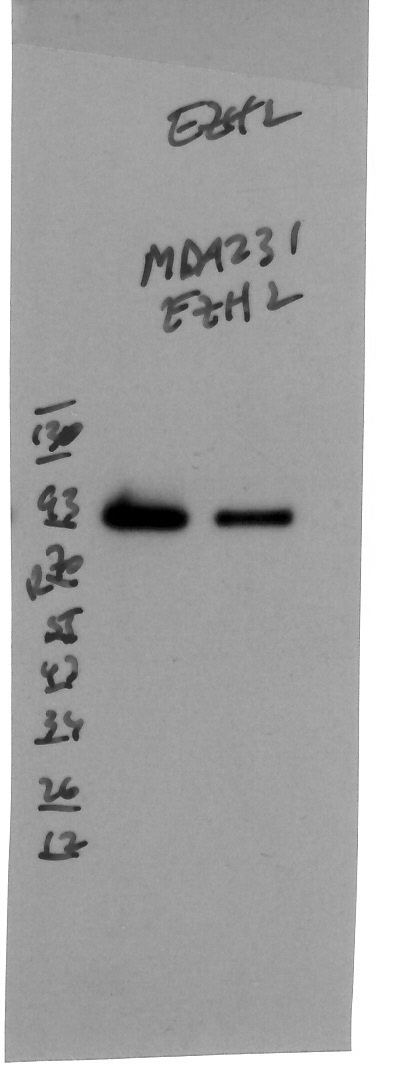

Supplement: Supplementary file 1 [file cancers-11-02039-s001.zip › cancers-631322-suppl-final2/Fig3_IB/EZH2_MDAMD 231 In vivo.tif]

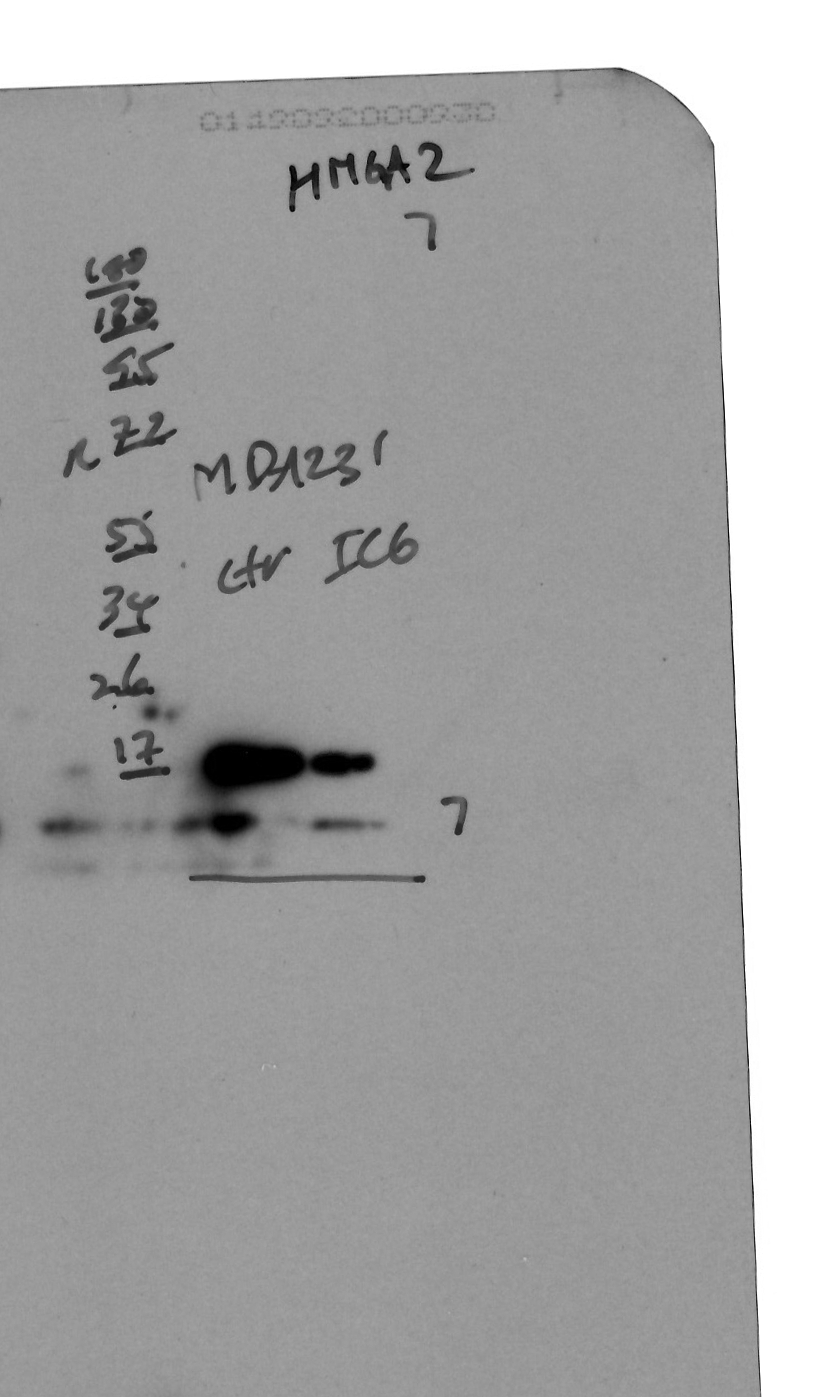

Supplement: Supplementary file 1 [file cancers-11-02039-s001.zip › cancers-631322-suppl-final2/Fig3_IB/HMGA2_MDAMD 231 In vivo.tif]

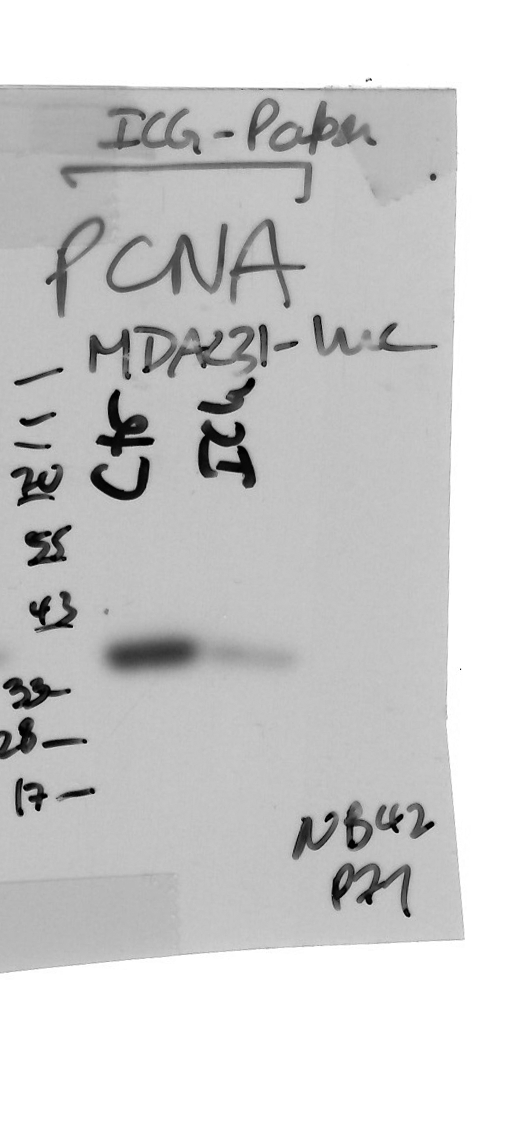

Supplement: Supplementary file 1 [file cancers-11-02039-s001.zip › cancers-631322-suppl-final2/Fig3_IB/PCNA MDAMD 231 In vivo_v2.tif]

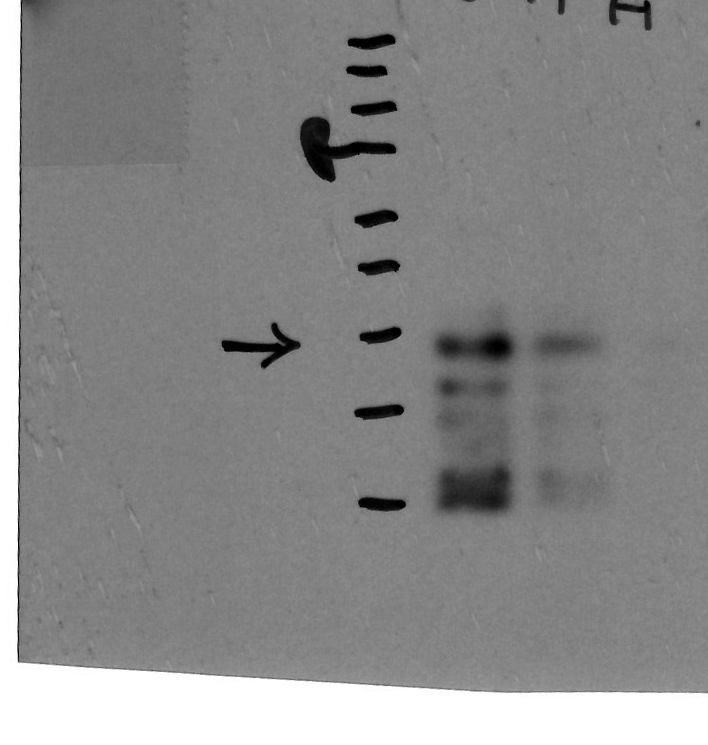

Supplement: Supplementary file 1 [file cancers-11-02039-s001.zip › cancers-631322-suppl-final2/Fig3_IB/SNAI_MDAMD 231 In vivo.tif]

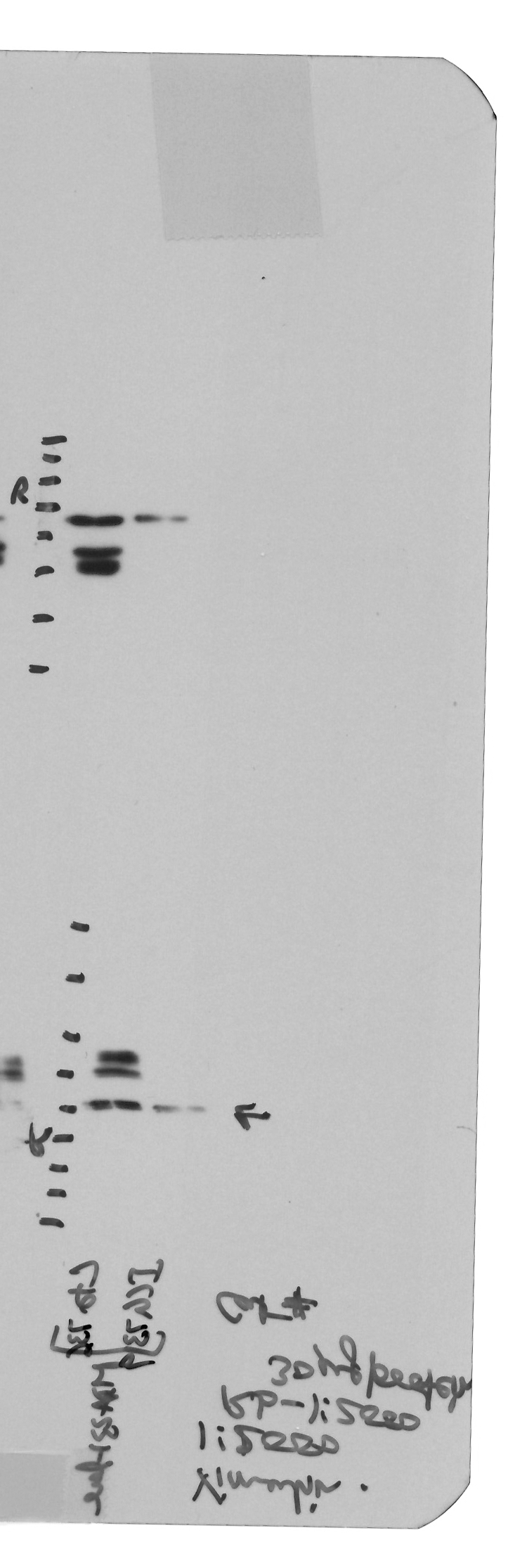

Supplement: Supplementary file 1 [file cancers-11-02039-s001.zip › cancers-631322-suppl-final2/Fig3_IB/Vimentin MDAMD 231 In vivo.tif]

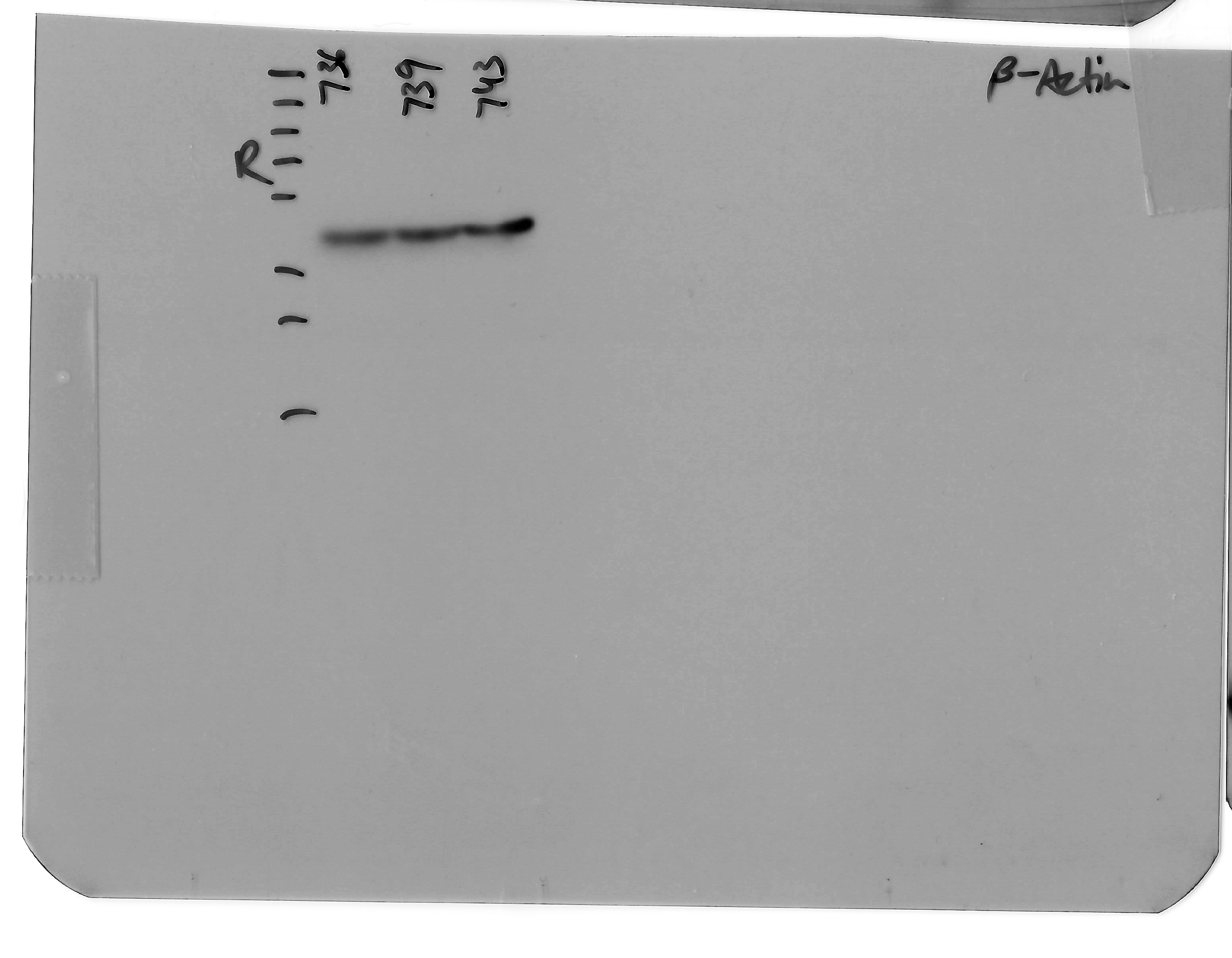

Supplement: Supplementary file 1 [file cancers-11-02039-s001.zip › cancers-631322-suppl-final2/Fig4_IB/Actin HCI-10_ In vivo.tif]

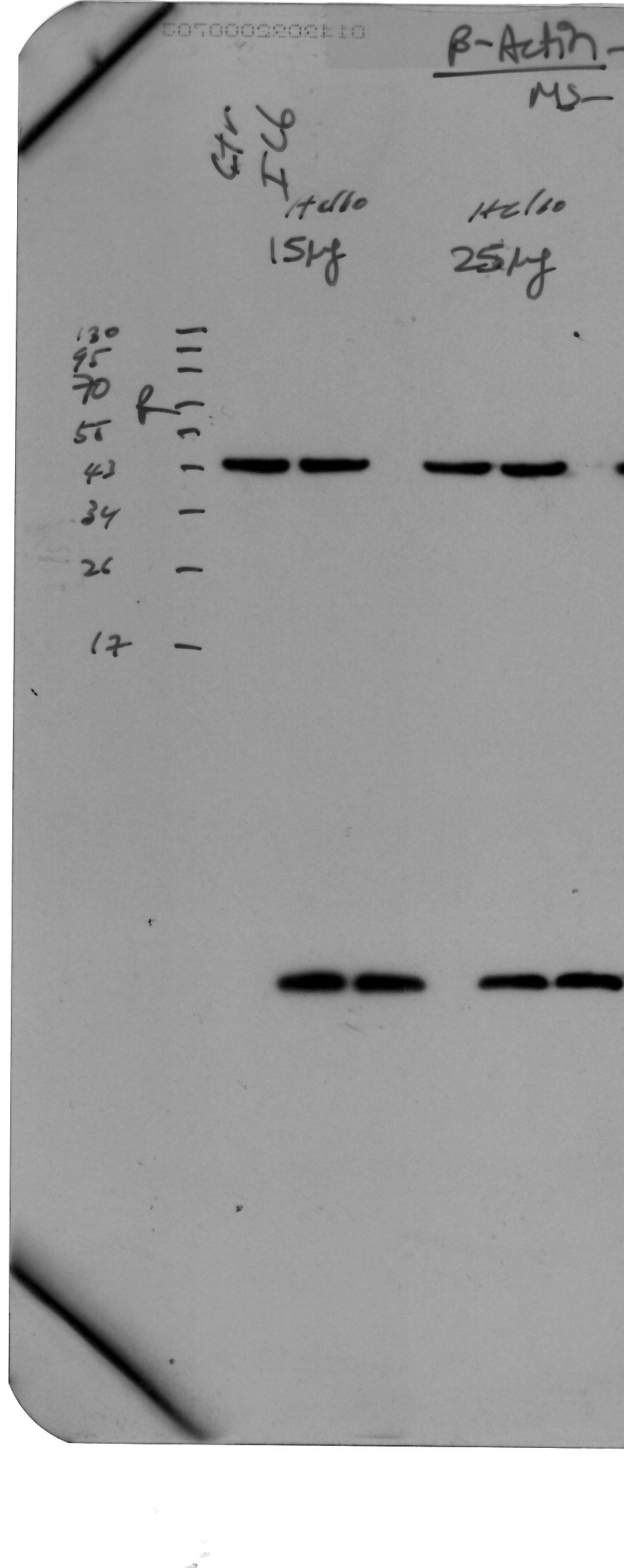

Supplement: Supplementary file 1 [file cancers-11-02039-s001.zip › cancers-631322-suppl-final2/Fig4_IB/Actin HCI-10_ In vivov2 .tif]

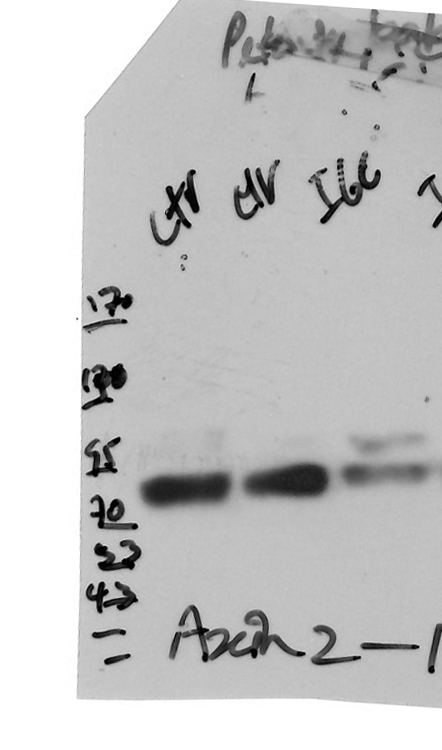

Supplement: Supplementary file 1 [file cancers-11-02039-s001.zip › cancers-631322-suppl-final2/Fig4_IB/AXIN2_HCI-10_ In vivo_v2.tif]

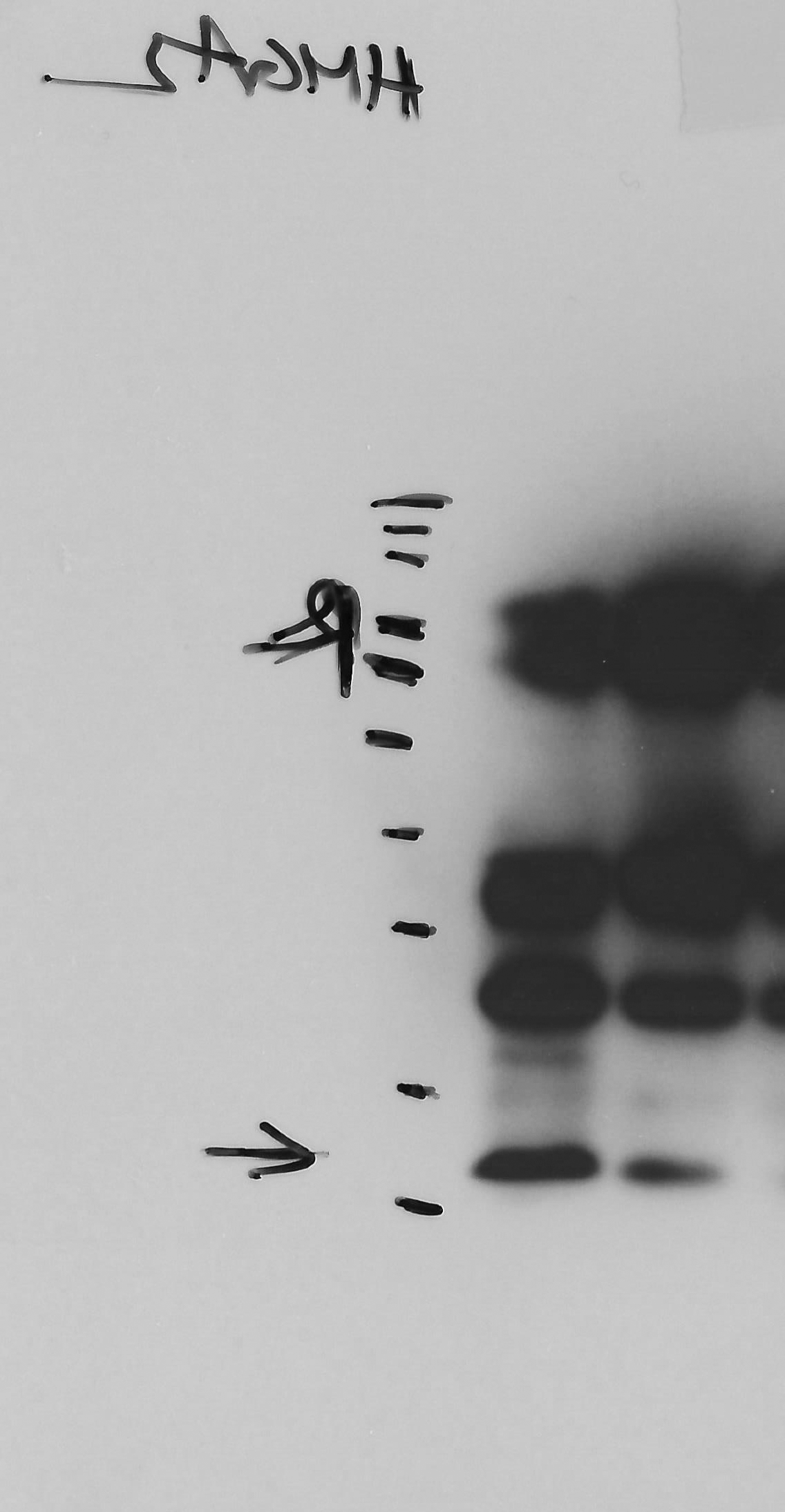

Supplement: Supplementary file 1 [file cancers-11-02039-s001.zip › cancers-631322-suppl-final2/Fig4_IB/HMGA2 HCI-10_ In vivo.tif]

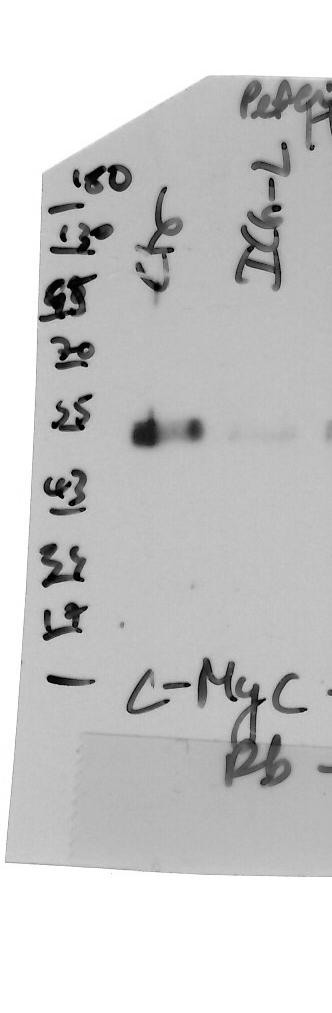

Supplement: Supplementary file 1 [file cancers-11-02039-s001.zip › cancers-631322-suppl-final2/Fig4_IB/Myc HCI-10_ In vivo.tif]

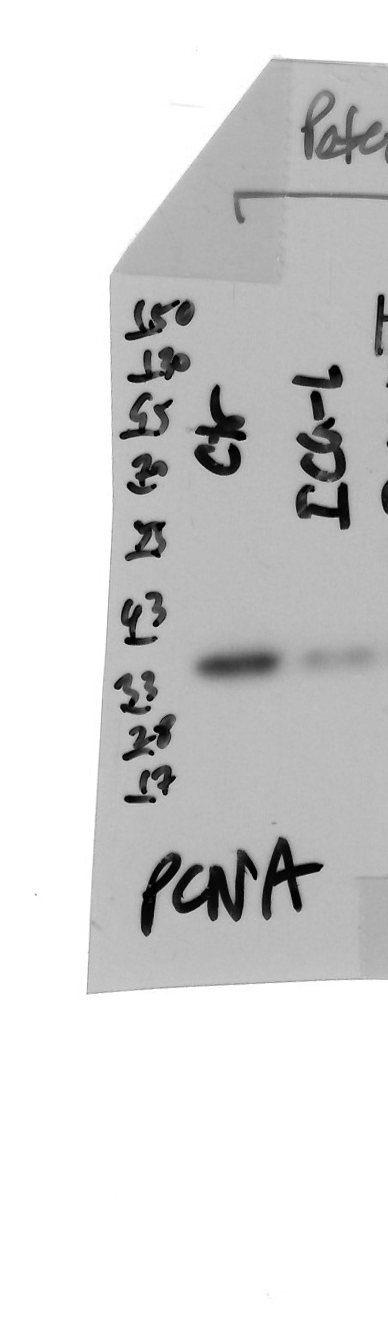

Supplement: Supplementary file 1 [file cancers-11-02039-s001.zip › cancers-631322-suppl-final2/Fig4_IB/PCNA HCI-10_ In vivo.tif]

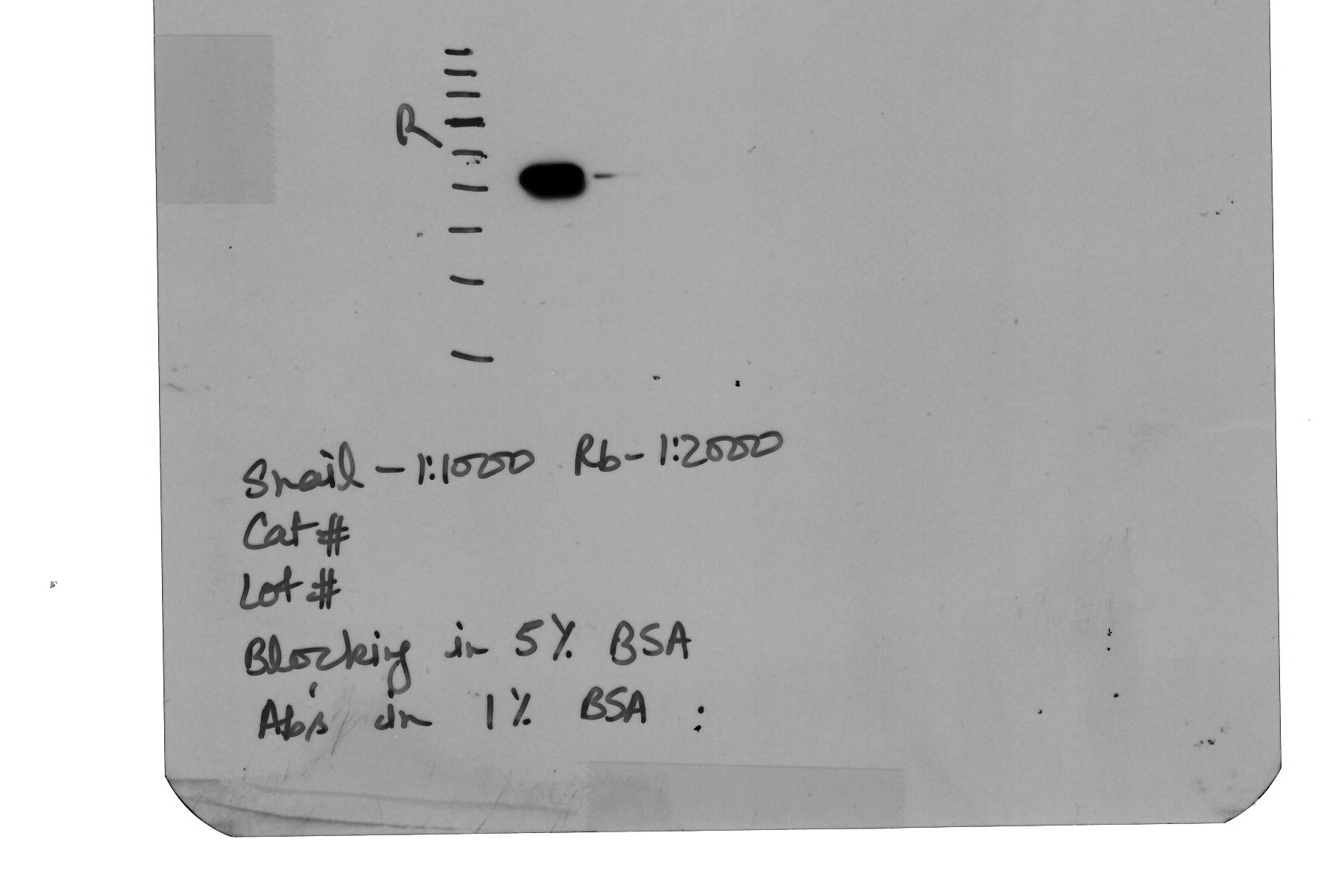

Supplement: Supplementary file 1 [file cancers-11-02039-s001.zip › cancers-631322-suppl-final2/Fig4_IB/Snail HCI-10_ In vivo.tif]

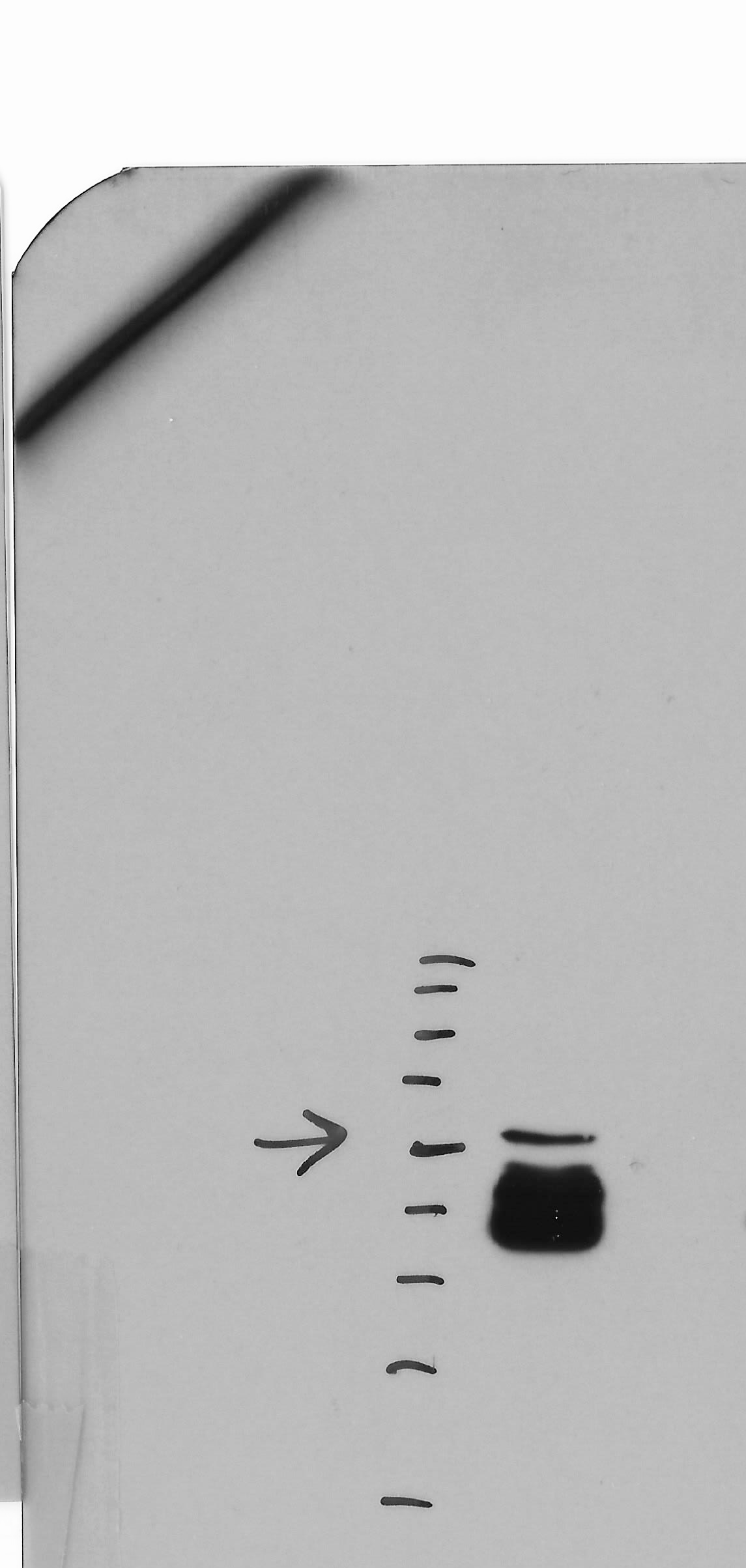

Supplement: Supplementary file 1 [file cancers-11-02039-s001.zip › cancers-631322-suppl-final2/Fig4_IB/Vimentin HCI-10 in vivo.tif]

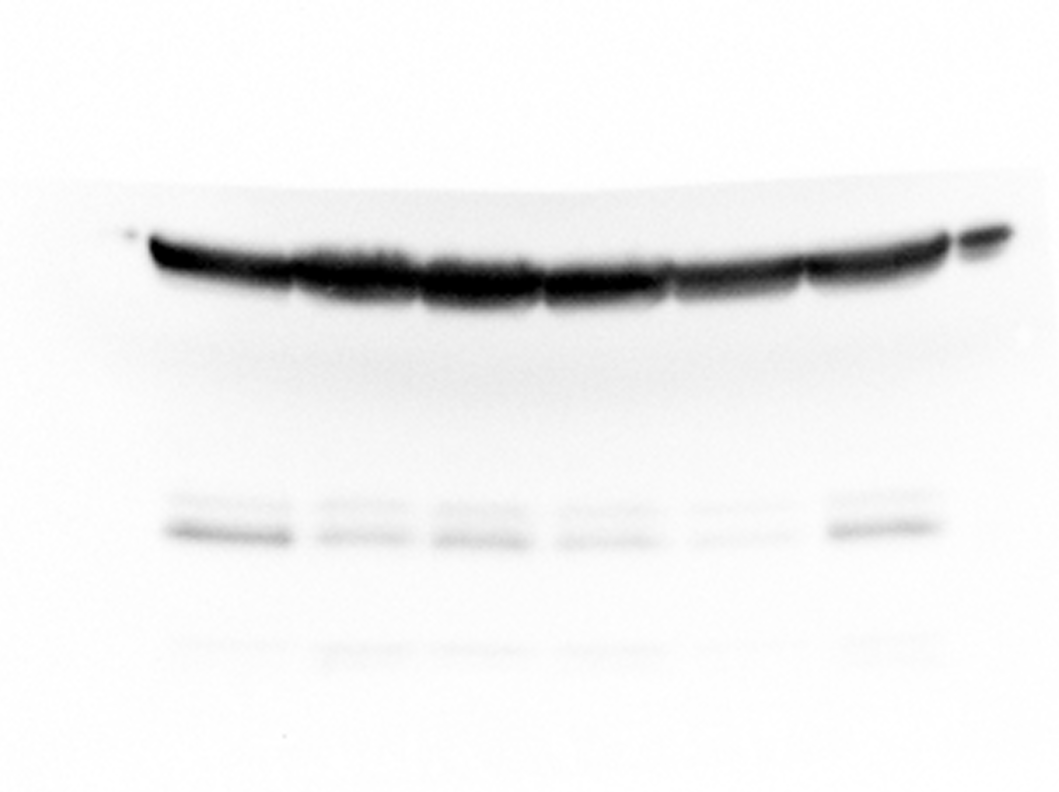

Supplement: Supplementary file 1 [file cancers-11-02039-s001.zip › cancers-631322-suppl-final2/Figure 2 IB/Fig 2_MDA231_tubulin.tif]

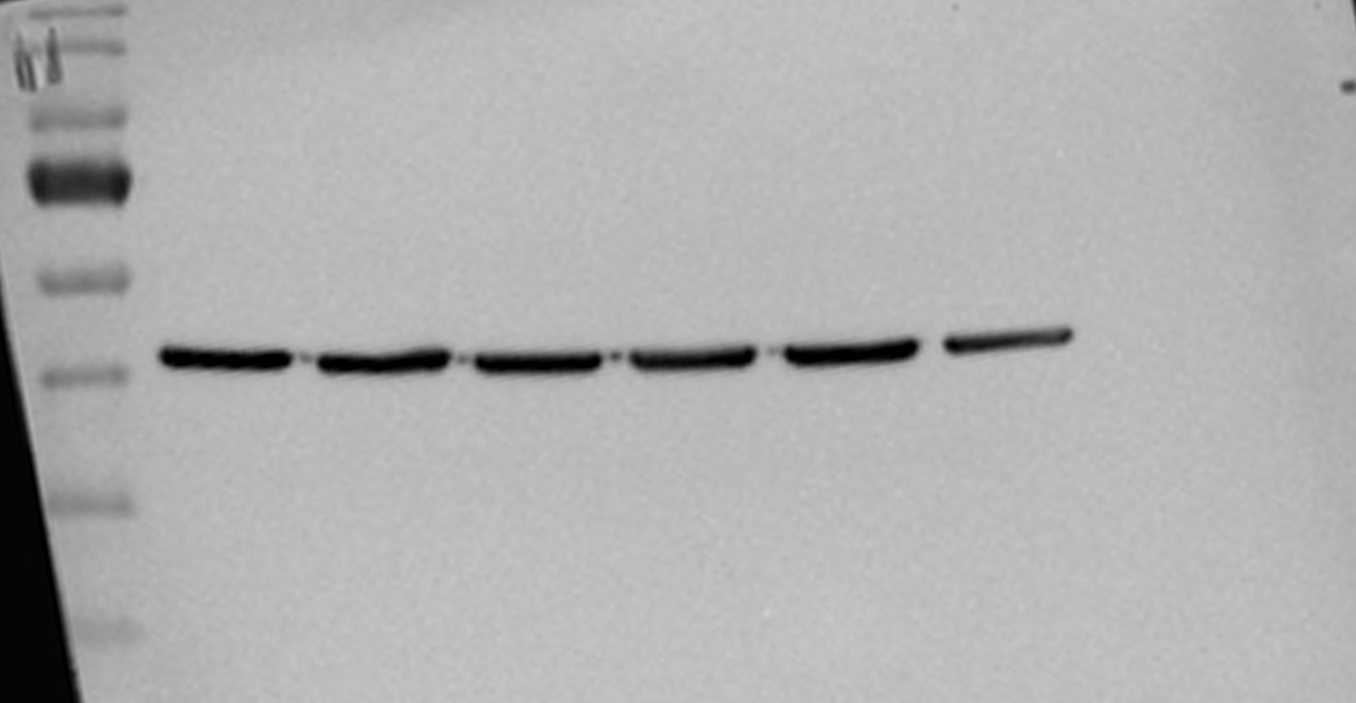

Supplement: Supplementary file 1 [file cancers-11-02039-s001.zip › cancers-631322-suppl-final2/Figure 2 IB/Fig2_ HCI-10_Actin.tif]

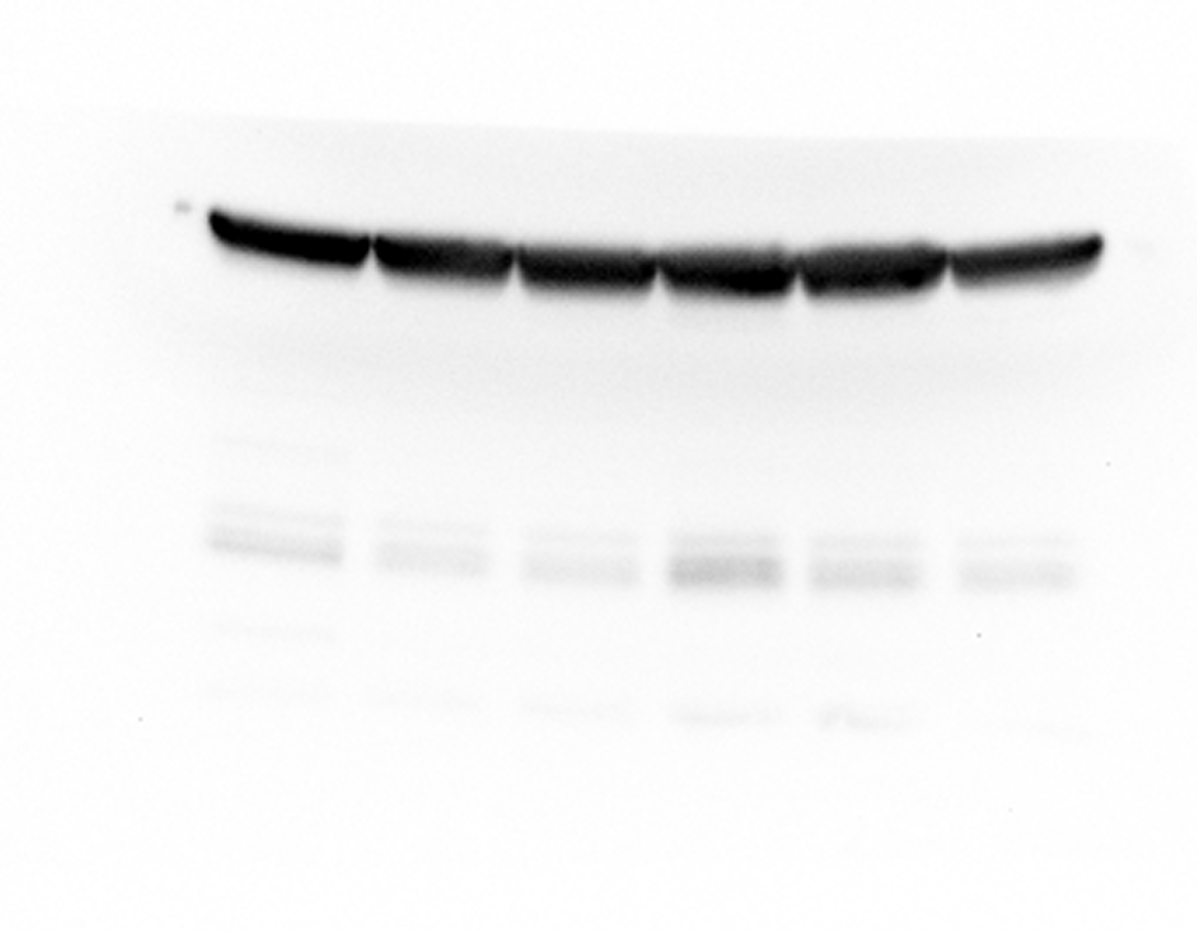

Supplement: Supplementary file 1 [file cancers-11-02039-s001.zip › cancers-631322-suppl-final2/Figure 2 IB/Fig2_ HCI-10_Tubulin.tif]

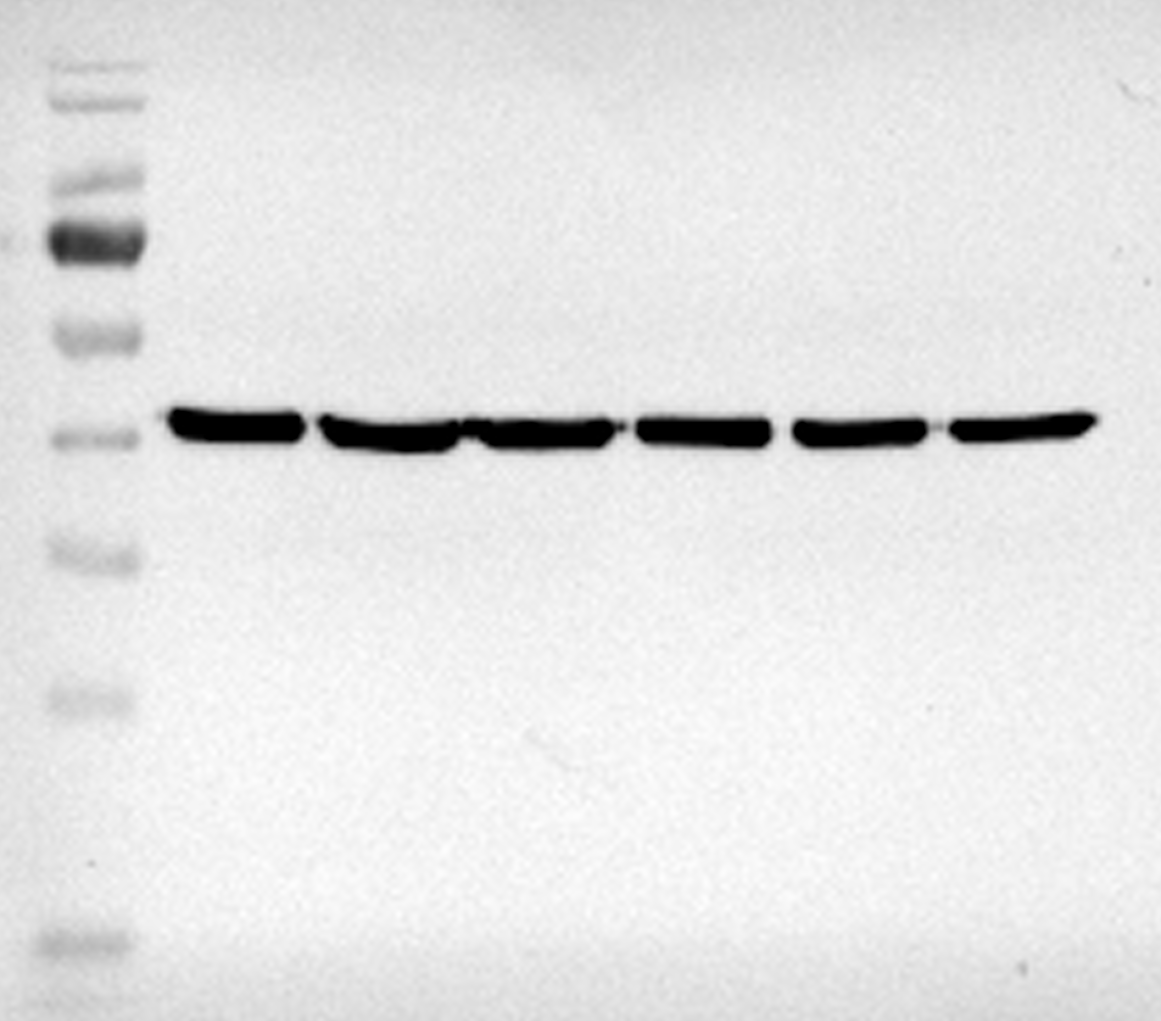

Supplement: Supplementary file 1 [file cancers-11-02039-s001.zip › cancers-631322-suppl-final2/Figure 2 IB/Fig2_ MDA231_Actin.tif]

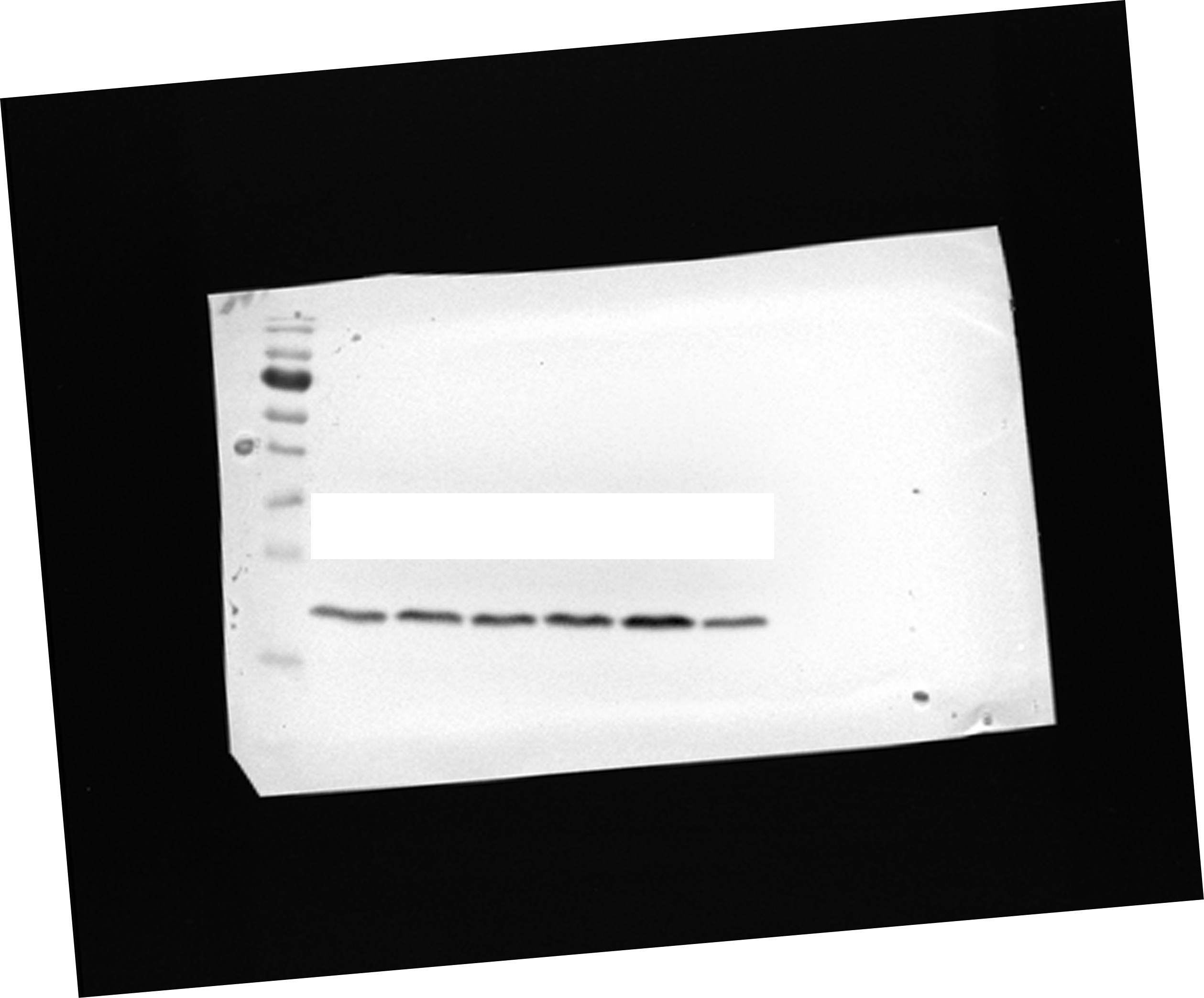

Supplement: Supplementary file 1 [file cancers-11-02039-s001.zip › cancers-631322-suppl-final2/Figure 2 IB/Fig2_HCL10_BAX.tif]

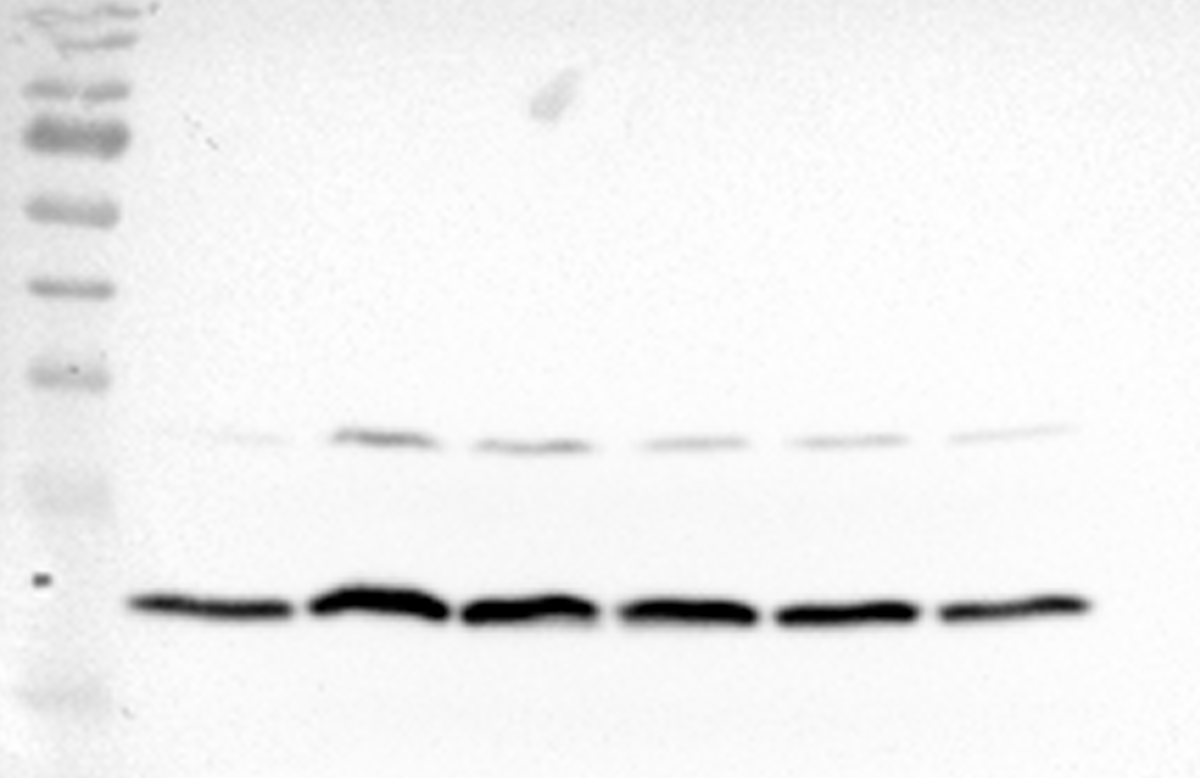

Supplement: Supplementary file 1 [file cancers-11-02039-s001.zip › cancers-631322-suppl-final2/Figure 2 IB/Fig2_MDA231_BAX.tif]
